# MT1-MMP inhibition rejuvenates ageing brain and rescues cognitive deficits in obesity

**DOI:** 10.1038/s41421-025-00825-w

**Published:** 2025-09-23

**Authors:** Pallavi Asthana, Liguo Li, Lin Lu, Jiayan Wu, Shuo Zhang, Ningning Li, Sheung Kin Ken Wong, Susma Gurung, Yijing Zhang, Yuwan Lin, Yufeng Peng, Zongtang Xu, Kui Ming Chan, Lixiang Zhai, Aiping Lyu, Zhao-Xiang Bian, Xin Ge, Ashok Iyaswamy, Min Li, Ya Su, Zhongjun Zhou, Pingyi Xu, Hoi Leong Xavier Wong

**Affiliations:** 1 School of Chinese Medicine, Hong Kong Baptist University, Hong Kong SAR, China; 2 Institute of Rehabilitation Medicine, Henan Academy of Innovations in Medical Science, Zhengzhou, Henan China; 3 Huanghe University of Science and Technology, Zhengzhou, Henan China; 4 Department of Neurology, the First Affiliated Hospital, Guangzhou Medical University, Guangzhou, Guangdong China; 5 Key Laboratory of Advanced Interdisciplinary Studies, The First Affiliated Hospital of Guangzhou Medical University, Guangzhou, Guangdong China; 6 Zhejiang Provincial Key Laboratory of Pancreatic Disease, the First Affiliated Hospital, Zhejiang University School of Medicine, Hangzhou, Zhejiang China; 7 MOE Joint International Research Laboratory of Pancreatic Diseases, the First Affiliated Hospital, Zhejiang University School of Medicine, Hangzhou, Zhejiang China; 8 Tomas Lindahl Nobel Laureate Laboratory, The Seventh Affiliated Hospital, Sun Yat-Sen University, Shenzhen, Guangdong China; 9 School of Biomedical Sciences, Li Ka Shing Faculty of Medicine, The University of Hong Kong, Hong Kong SAR, China; 10 Department of Biomedical Sciences, City University of Hong Kong, Hong Kong SAR, China; 11 Centre for Chinese Herbal Medicine Drug Development Limited, Hong Kong Baptist University, Hong Kong SAR, China; 12 Institute of Molecular Medicine, University of Texas Health Science Center at Houston, Houston, TX USA; 13 Mr. & Mrs. Ko Chi-Ming Centre for Parkinson’s Disease Research, School of Chinese Medicine, Hong Kong Baptist University, Hong Kong SAR, China; 14 Department of Biochemistry, Karpagam Academy of Higher Education, Coimbatore, India; 15 Golden Meditech Centre for NeuroRegeneration Sciences, School of Chinese Medicine, Hong Kong Baptist University, Hong Kong SAR, China; 16 Institute for Research and Continuing Education, Hong Kong Baptist University, Shenzhen, Guangdong China

**Keywords:** Ageing, Mechanisms of disease, Proteolysis

## Abstract

Obesity has been linked to an increased risk of cognitive impairment and dementia in later life. Although aging and obesity are both associated with cognitive decline, it remains unclear how they interact to affect cognitive function across the lifespan and how brain function might mediate their relationship with cognition. Our previous findings and other studies have shown that membrane type 1-matrix metalloproteinase (MT1-MMP/MMP14), which increases with age, regulates energy homeostasis. Inhibiting MT1-MMP improves insulin sensitivity, reduces body fat, and lowers serum cholesterol. Here, we demonstrate that MT1-MMP links neuroinflammation to cognitive decline in aging and obesity. Inflammatory responses in the brain increase MT1-MMP activation in the hippocampus of both mice and humans. Activation of hippocampal MT1-MMP alone can trigger cognitive decline and synaptic impairment independently of neuroinflammation. Conversely, ablation of MT1-MMP in the hippocampus reverses cognitive decline and improves synaptic plasticity in aging and obesity. Pharmacological inhibition of MT1-MMP, through an orally administered brain-penetrant inhibitor or targeted delivery of a neutralizing antibody to the hippocampus, improves memory and learning in aged and obese mice without toxicity. Mechanistically, MT1-MMP proteolytically inactivates G-protein-coupled receptor 158 (GPR158), a hippocampal receptor for osteocalcin (OCN) that is important for the maintenance of cognitive integrity, thus suppressing the ability of the OCN-GPR158 axis to promote cognition in aging and obesity. These findings suggest a new mechanism underlying hippocampal dysfunction and reveal the potential for treating multiple age-related diseases, including neurodegenerative disorders, obesity, diabetes, and atherosclerosis, with a single MT1-MMP-blocking agent.

## Introduction

Aging leads to dramatic changes in cognitive and neuronal function, the decline of which contributes to the development of neurological disorders^1^. Because the elderly population is growing, it is important to identify a means for maintaining cognitive integrity by protecting against, or even counteracting the aging process.

How aging leads to cognitive decline remains largely unclear. Aging is associated with neuroinflammation and metabolic disorders, both of which contributes to cognitive decline and various neurodegenerative diseases. Although aging and obesity are both associated with cognitive decline, it remains unclear how they interact together to affect cognitive function across the life span and how brain function might mediate their relationship on cognition.

In recent years, increasing evidence has suggested that neuroinflammation is a major contributor to cognitive impairment^2–4^. During aging, chronic low-grade inflammation occurs in the absence of active infection, contributing to a wide range of age-associated diseases, such as neurodegenerative diseases, obesity, and diabetes. Inflammaging in the brain is characterized by increased levels of proinflammatory cytokines, aberrant activation of astrocytes and microglia, and increased oxidative stress^2–4^. Neuroinflammation can directly damage neurons, impair synaptic plasticity, and disrupt neuronal networks, leading to cognitive decline and functional impairments^5–7^. Given the multiple functions of many inflammatory factors, the central inflammatory meditator that links neuroinflammation to cognitive impairment remains undefined. The molecular mechanism by which neuroinflammation drives cognitive dysfunctions is also largely unclear. Understanding the relationship between neuroinflammation and aging is crucial for developing therapeutic strategies to mitigate age-related neurological disorders and promote healthy aging.

The extracellular matrix (ECM) is a complex network of molecules that provides structural and biochemical support to surrounding cells in tissues and organs. In the brain, the ECM is particularly important for maintaining the structural integrity of neural tissues and homeostatic neuronal processes, including synaptic functions^8,9^. ECM remodeling is regulated by various enzymes, especially matrix metalloproteinases (MMPs)^10^. Although MMPs have been implicated in regulating synaptic remodeling and maintaining the blood‒brain barrier (BBB) by altering ECM components^11,12^, little is known about the effects of MMPs in the context of cognitive aging. MT1-MMP/MMP14, a membrane-bound MMP, is one of the most investigated MMPs. Physiologically, MMP14 cleaves a wide range of substrates, ranging from ECM to growth factor receptors, in a tissue-specific and contextual manner^13,14^. Our recent findings, along with those of other studies, demonstrate that MT1-MMP is a key regulator of energy homeostasis and that a series of proteolytic events mediated by MT1-MMP contribute to the pathogenesis of multiple age-associated metabolic disorders, including obesity, diabetes, and atherosclerosis^15–17^. In particular, proteolytic cleavage of the anorectic receptor GDNF family receptor alpha-like (GFRAL) by neuronal MT1-MMP regulates the satiety center in the brainstem, exerting non-homeostatic control of food intake and body weight^15^. In addition, elevated levels of MMP14 and aberrant MMP14 activity have been implicated in various age-related neurological conditions associated with cognitive impairment, such as Alzheimer’s disease and traumatic brain injury^18,19^. Notably, a recent study has revealed that MMP14 is associated with dementia in middle-aged adults in a human cohort with 10,981 participants. MT1-MMP is constitutively shed via proteolytic cleavage by A Disintegrin and Metalloproteinase (ADAM) family metalloproteinases, resulting in detectable quantities of soluble MT1-MMP (sMT1-MMP) in the plasma^20–22^. Our previous studies revealed a significant increase in sMT1-MMP levels with increasing age in mice, cynomolgus macaques, and humans^16,17^. Further research is needed to fully elucidate the relationship between MT1-MMP and cognitive impairment, given the strong association between MT1-MMP activation, metabolic dysfunctions, and age-related neurological disorders, and to explore the potential therapeutic implications of targeting MT1-MMP in the context of cognitive aging and other neurological disorders associated with metabolic disorders.

## Results

### Age-related neuroinflammation drives MT1-MMP activation in the hippocampus

**Fig. 1:**
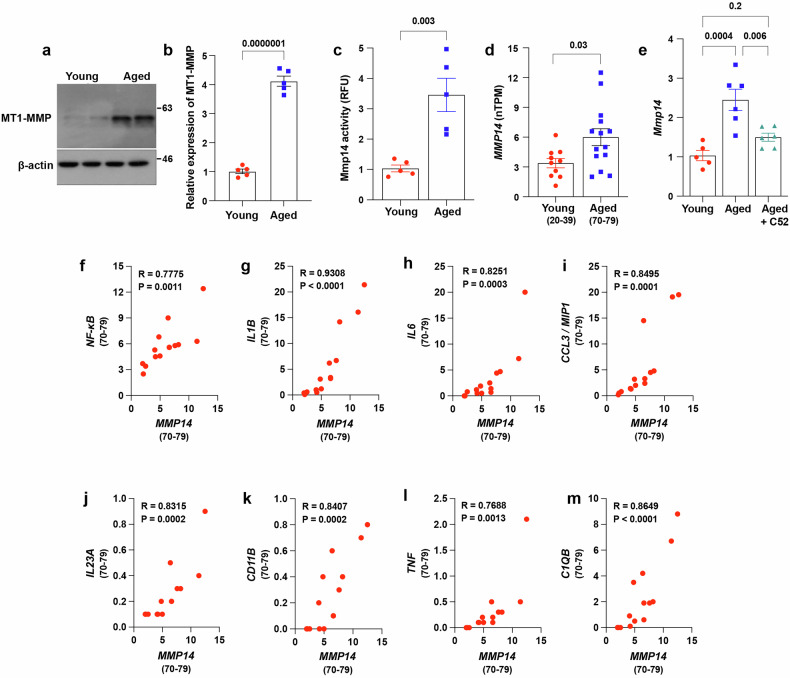
Age-related neuroinflammation drives MT1-MMP activation in the hippocampus. **a** Immunoblotting of MT1-MMP from hippocampal lysates of 2-month-old (young) and 18-month-old (aged) mice (*n* = 2 mice per group). **b** Quantification of MT1-MMP expression relative to β-actin loading control (*n* = 5 mice per group). Two-tailed unpaired Student’s *t*-test. **c** MMP14 activity was measured as relative fluorescence units (RFU) in the hippocampus sample of young and aged mice. Two-tailed unpaired Student’s *t*-test (*n* = 5 mice per group). **d** *MMP14* normalized transcript per million (nTPM) data from adult genotype tissue expression (GTEx) portal and comparative analysis between young and aged human hippocampus tissue. Two-tailed unpaired Student’s *t*-test. **e** Hippocampal *Mmp14* mRNA levels by qPCR in young, aged, and C52-treated aged mice. One-way ANOVA followed by Tukey’s multiple comparisons test. (*n* = 5 young, *n* = 6 aged, *n* = 6 aged + C52 mice). **f**–**m** Correlation analysis between *MMP14* and pro-inflammatory factors *NF-κB* (**f**), *IL-1B* (**g**), *IL-6* (**h**), *CCL3* (**i**), *IL23A* (**j**), *CD11B* (**k**), *TNF* (**l**) and *C1QB* (**m**) from the RNA sequencing data from human hippocampus tissues obtained through GTEx portal. Data are expressed as mean ± SEM, and each data point represents individual mouse/human.

To understand the regulatory role of MT1-MMP in cognitive integrity, we first examined changes in MT1-MMP expression in the brains of 18-month-old aged mice. With aging, the level of MT1-MMP in the hippocampus, a brain region that is responsible for learning and memory and highly susceptible to the detrimental effects of aging, significantly increased in mice (Fig. 1a, b). In line with increased MMP14 expression, an MMP14-specific activity assay revealed increased MT1-MMP activity in the hippocampus in aged mice (Fig. 1c). To investigate further the physiological relevance of MMP14 upregulation in human aging, we analyzed reference datasets containing the hippocampal transcriptomes of both young and aged subjects. *MMP14* expression in the hippocampus was consistently elevated in older adults (Fig. 1d), suggesting that the increased activation of hippocampal MT1-MMP during aging is conserved across vertebrates. Given the strong association between neuroinflammation and cognitive impairment in aging, we reasoned that age-related neuroinflammation may contribute to the upregulation of MT1-MMP. Chronic inflammation is a hallmark of brain aging that is characterized by the increased expression of proinflammatory genes and initiation of the complement cascade. We examined neuroinflammatory markers in the brains of 18-month-old aged mice after the systemic administration of compound 52 (C52), a brain-penetrant inhibitor of prostaglandin/E2 signaling that suppresses neuroinflammation and restores cognitive function during aging^23^. Treatment of aged mice with C52 for one month restored the expression of proinflammatory factors, including *Tnf, Cd11b, Il1b and Nfkb1* (Supplementary Fig. S1), and the level of *Mmp14* (Fig. 1e) in the hippocampus to the corresponding levels in two-months-old (young) mice, suggesting that neuroinflammation may drive the upregulation of MT1-MMP during aging. To further confirm this finding, we analyzed the association between MMP14, pro-inflammatory cytokines, and genes that are actively involved in neuroinflammation in aged human brains. The expression of *MMP14* was positively correlated with a panel of neuroinflammatory markers including *NF-kB, IL1B, IL6, CCL3, IL23A, CD11B, IL13, TNF, and C1QB* (Fig. 1f–m), reinforcing the notion that neuroinflammation contributes to MT1-MMP activation in human aging.

### MT1-MMP blockade reverses cognitive aging without altering neuroinflammation

**Fig. 2:**
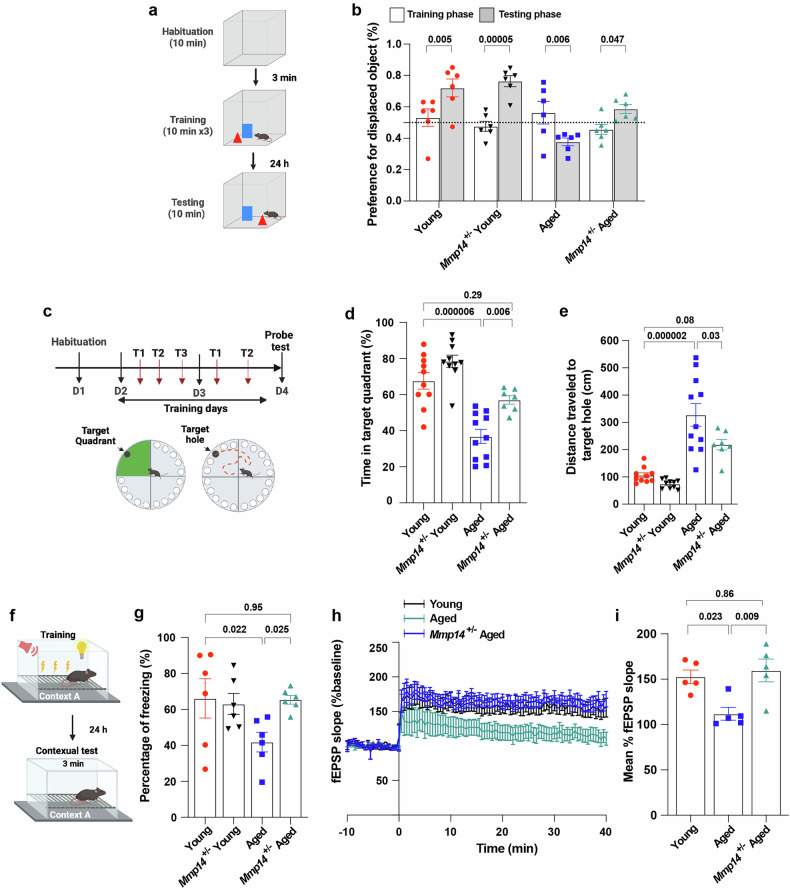
Systemic MT1-MMP depletion alleviates aging-associated cognitive impairment. **a** Schematic diagram of object location test. **b** Time spent exploring the displaced object expressed as the preference for the displaced object (%) from 2-month-old (young) to 18-month-old (aged) WT and *Mmp14*^*+/−*^ mice. One-way ANOVA followed by Fisher’s LSD post hoc test (*n* = 6 mice per group). **c** Schematic diagram of Barnes maze test. **d**, **e** Percentage of total time spent in the target quadrant (**d**) and distance traveled to the target hole (**e**) in Barnes maze test. One-way ANOVA followed by Tukey’s multiple comparisons test (*n* = 10 young, *n* = 10 *Mmp14*^*+/−*^ young, *n* = 11 aged, *n* = 7 *Mmp14*^*+/−*^ aged mice). **f** Schematic diagram of contextual fear conditioning test. **g** Percentage of freezing time during a contextual fear conditioning test performed by young and aged WT and *Mmp14*^*+/−*^ mice. One-way ANOVA followed by Fisher’s LSD post hoc test (*n* = 6 mice per group). **h**, **i** LTP in the CA1 hippocampal region quantified as the change in fEPSP (**h**) and the average of fEPSP (**i**) from young and aged WT and *Mmp14*^*+/−*^ mice. One-way ANOVA followed by Tukey’s multiple comparisons test (*n* = 5 mice per group). Data are expressed as mean ± SEM, and each data point represents individual mouse.

To investigate whether MT1-MMP regulates cognitive aging, the role of MT1-MMP in age-associated cognitive function was examined in *Mmp14* haploinsufficient (*Mmp14*^*+/−*^*)* mice, which do not display gross postnatal phenotypes^24–26^. Given that mammalian brain ageing is accompanied by memory loss and reduced synaptic plasticity, but not significantly by neuronal loss, behavioral tests are typically used in the evaluation of learning and memory in the physiological ageing. We tested the performance of the mice in the object location memory task (Fig. 2a, b) and the Barnes maze task (Fig. 2c–e), two standard tests used to evaluate hippocampus-dependent spatial memory. In both tasks, compared with 2-month-old (young) mice, 18-month-old (aged) mice exhibited significant deficits in hippocampus-dependent learning and performance (Fig. 2b, d, e). Although no overt changes in physical activity were noted (Supplementary Fig. S2a–d), the performance of aged *Mmp14*^*+/−*^ mice in both tasks was markedly improved and became indistinguishable from that of young mice of either genotype (Fig. 2b, d, e). In addition, the freezing level was significantly greater in 18-month-old *Mmp14*^*+/−*^ mice during the hippocampus-dependent contextual memory task (Fig. 2f, g), indicating improved contextual memory in *Mmp14*^*+/−*^ mice during aging. Moreover, we evaluated hippocampal synaptic plasticity in 18-month-old aged *Mmp14*^*+/−*^ mice using electrophysiological recordings and found that these mice showed robust improvements in long-term potentiation (LTP) (Fig. 2h, i), a key determining factor in learning and memory that deteriorates with age. To investigate whether the cognitive rescue observed in aged *Mmp14*^*+/−*^ mice was associated with altered inflammatory responses in the brain, we examined the hippocampal levels of proinflammatory cytokines, including Il-17A, Il3, Il6, MIP1, Il13, Il22 and Il23, in young (2-month-old) and aged (18-month-old) wild-type (WT) and *Mmp14*^*+/−*^ mice. Surprisingly, the levels of all of these factors in the hippocampus were elevated to a similar extent in aged WT and *Mmp14*^*+/−*^ mice (Supplementary Fig. S2e–k). Flow cytometry analyses revealed that there were also no changes in microglia activation in the hippocampus of 18-month-old aged *Mmp14*^*+/−*^ mice (Supplementary Fig. S2l–n). These data suggest that *Mmp14* haploinsufficiency does not modulate age-associated neuroinflammation.

Exposure to pro-aging circulating factors present in the blood of aged mice induces neuroinflammation and triggers age-related cognitive decline in young rodents^27,28^. To determine whether MT1-MMP mediates the detrimental effects of aged plasma on the hippocampus, we infused aged plasma into young (3-month) WT and *Mmp14*^*+/−*^ mice (Supplementary Fig. S3a). We found that repeated infusions of plasma from 24-month-old aged mice, but not 2-month-old young mice, for four weeks induced a significant increase in MT1-MMP expression in the hippocampus and elicited cognitive deficits in 2-month-old young WT mice. However, the damaging effects of aged plasma on cognition were absent in 2-month-old young *Mmp14*^*+/−*^ mice (Supplementary Fig. S3b–d), suggesting that MT1-MMP serves as a key mediator linking systemic aging signals to hippocampal dysfunction and cognitive impairment. Thus, a systemic reduction in MT1-MMP in aged mice can prevent the decline of memory, learning, and synaptic plasticity without significantly altering age-related neuroinflammation.

**Fig. 3:**
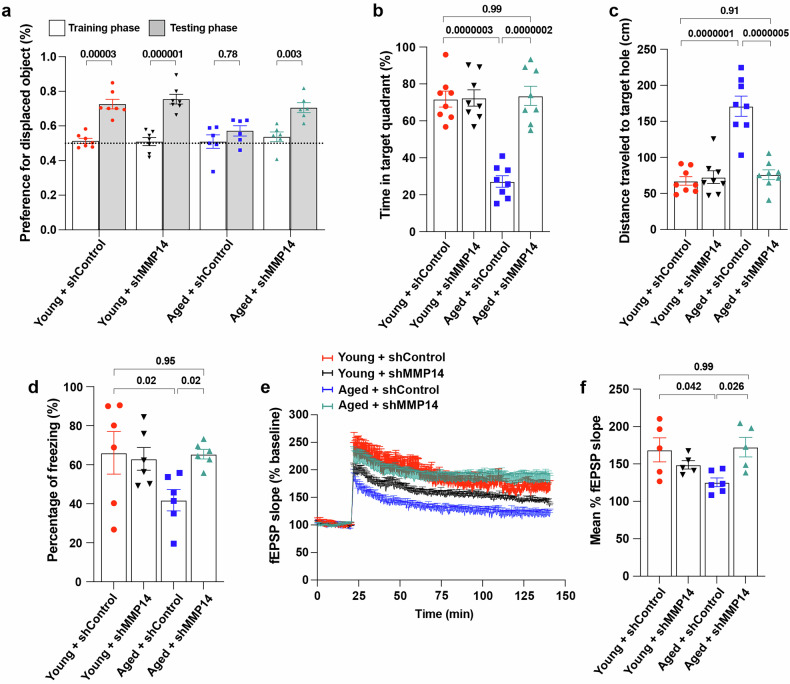
Selective ablation of MT1-MMP in the hippocampus reverses cognitive impairment. **a** Preference for the displaced object in object location task by 2-month-old (young) and 18-month-old (aged) mice injected with shRNA targeting *Mmp14* (shMMP14*)* or scrambled shRNA (shControl) in the DG region of the hippocampus. One-way ANOVA followed by Tukey’s multiple comparisons test (*n* = 7 young + shControl, *n* = 7 young + shMMP14, *n* = 6 aged + shControl, *n* = 6 aged + shMMP14). **b**, **c** Percentage of total time spent in the target quadrant (**b**) and distance traveled to the target hole (**c**) in the Barnes maze test. One-way ANOVA followed by Tukey’s multiple comparisons test (*n* = 8 mice per group). **d** Percentage of freezing time during a contextual fear conditioning test. One-way ANOVA followed by Fisher’s LSD post hoc test (*n* = 6 mice per group). **e**, **f** Change in fEPSP in the hippocampal CA1 for LTP analysis (**e**) and the average of fEPSP (**f**). One-way ANOVA followed by Tukey’s multiple comparisons test (*n* = 5 young + shControl, *n* = 5 young + shMMP14, *n* = 6 aged + shControl, *n* = 5 aged + shMMP14). Data are expressed as mean ± SEM, and each data point represents individual mouse.

We then tested whether specific knockdown of MT1-MMP in the hippocampus might restore cognitive function to a level similar to that observed in aged *Mmp14*^*+/−*^ mice. To specifically deplete MT1-MMP in the hippocampus, we injected lentiviruses expressing either shRNA targeting *Mmp14* or scrambled shRNA as a control in the dorsal hippocampus of 18-month-old aged WT mice (Supplementary Fig. S4a). Consistent with the results obtained from *Mmp14*^*+/−*^ mice, depletion of hippocampal MT1-MMP in aged mice reversed hippocampus-dependent memory deficits in the object location memory task (Fig. 3a) and the Barnes maze task (Fig. 3b, c), restoring these functions to the levels observed in 2-month-old (young) mice. A similar result was observed when the mice were subjected to contextual fear conditioning (Fig. 3d). Moreover, electrophysiological recordings revealed that the knockdown of hippocampal MT1-MMP in aged mice restored LTP to a level similar to that observed in young mice, which was consistent with improvements in learning and memory (Fig. 3e, f). Notably, the induction of neuroinflammation in the hippocampus, marked by elevated levels of proinflammatory factors and increased microglia activation, in the aged mice was not altered by the knockdown of hippocampal MT1-MMP (Supplementary Fig. S4b–e). These results consistently show that the targeted ablation of hippocampal MT1-MMP can restore hippocampus-dependent memory function and synaptic plasticity to the levels observed during youth without significantly altering neuroinflammation, suggesting that MT1-MMP mediates neuroinflammation-induced cognitive dysfunction in aging.

### Depletion of hippocampal MT1-MMP rescues obesity-induced hippocampal dysfunction

Obesity is associated with systemic inflammation, leading to neuroinflammation and cognitive impairment^29^. To investigate whether hippocampal MT1-MMP similarly links systemic low-grade inflammation to cognitive decline in the context of obesity, we used a high-fat diet-induced (HFD) obesity mouse model for further studies. Our previous studies have shown that MT1-MMP controls non-homeostatic body weight by restraining the anorectic effect of growth differentiation factor 15 (GDF15)-mediated GFRAL signaling. Systemic inhibition of MT1-MMP or specific deletion of MT1-MMP in GFRAL^+^ brainstem neurons confers protections against diet-induced obesity in mice^15,17^. To avoid the influence of potential weight-loss effects conferred by the depletion of hippocampal MT1-MMP, WT mice at the age of 8 weeks were maintained on HFDs for 9 weeks, followed by the injection of lentiviruses expressing either shRNA targeting *Mmp14* (HFD/shMMP14) or scrambled shRNA (HFD/shControl) in the dorsal hippocampus (Supplementary Fig. S5a). Parallel groups of mice were maintained on chow diet as a control (CD/shControl). The mice were subjected to various behavior tests for assessing spatial memory functions for two weeks after lentiviral injections. In line with our previous findings^15^, HFD-induced obesity led to increased expression of hippocampal MT1-MMP, which was reversed by the lentivirus-mediated knockdown of MT1-MMP (Supplementary Fig. S5b). Similar weight gain was observed in HFD/shMMP14 and HFD/shControl mice (Supplementary Fig. S5c). Analyses of cytokines in the hippocampus and serum revealed that HFD consumption increased circulating levels of several pro-inflammatory cytokines, including IL1B, IL6, and TNFα, and this effect was comparable between HFD/shMMP14 and HFD/shControl mice (Supplementary Fig. S5d, e). HFD/shControl mice exhibited deficits in memory and cognition in both the object location memory task (Supplementary Fig. S5f), the Y-maze test (Supplementary Fig. S5g), and the water maze test (Supplementary Fig. S5h, i). The targeted depletion of hippocampal MT1-MMP restored memory functions in HFD mice to levels observed in CD/shControl mice without altering inflammatory responses in the hippocampus (Supplementary Fig. S5f–i). Moreover, electrophysiological recordings in brain slice preparations showed that consumption of HFD for 11 weeks impaired LTP in HFD/shControl mice, and this effect was abrogated in HFD/shMMP14 mice (Supplementary Fig. S5j, k). The results obtained from both the naturally aged model and the obesity model consistently reveal that MT1-MMP is a key determinant factor for chronic inflammation-induced cognitive impairment.

Growing evidence has shown that MMPs, especially MMP9, may contribute to cognitive impairment in neurodegenerative diseases through their involvement in blood-brain barrier (BBB) disruption^30^, a major driving factor for cognitive aging. To determine whether the restorative effects of MT1-MMP depletion on cognitive function were linked to improvements in BBB integrity, we injected young (2-month-old) and aged (18-month-old) WT and *Mmp14*^*+/−*^ mice with a 2% Evan’s dye and quantified the levels 24 h later. We did not observe any significant differences in Evan’s blue diffusion in the hippocampus (Supplementary Fig. S6a) and the expression of major genes involved in the maintenance of BBB permeability and architecture because of MT1-MMP depletion (Supplementary Fig. S6b–d), suggesting that MT1-MMP does not alter BBB integrity in aging and that the cognitive rescue conferred by MT1-MMP depletion in ageing is not related to the prevention of BBB disruption.

MT1-MMP is a major activator of other MMPs, including MMP2 and MMP9. To investigate whether other MMPs are involved in the regulation of cognitive integrity during aging, we assessed the performance of 18-month-old age-matched *Mmp2*^*−/−*^*, Mmp9*^*−/−*^, and *Mmp14*^*+/−*^ mice in the object location memory task (Supplementary Fig. S7a) and the Barnes maze task (Supplementary Fig. S7b, c). We found that only aged *Mmp14*^*+/−*^ mice exhibited significant improvements in learning and memory (Supplementary Fig. S7a–c), suggesting that age-related cognitive functions are specifically regulated by MT1-MMP.

### Pharmacological inhibition of MT1-MMP improves memory function and metabolism in both aged mice and obese mice

**Fig. 4:**
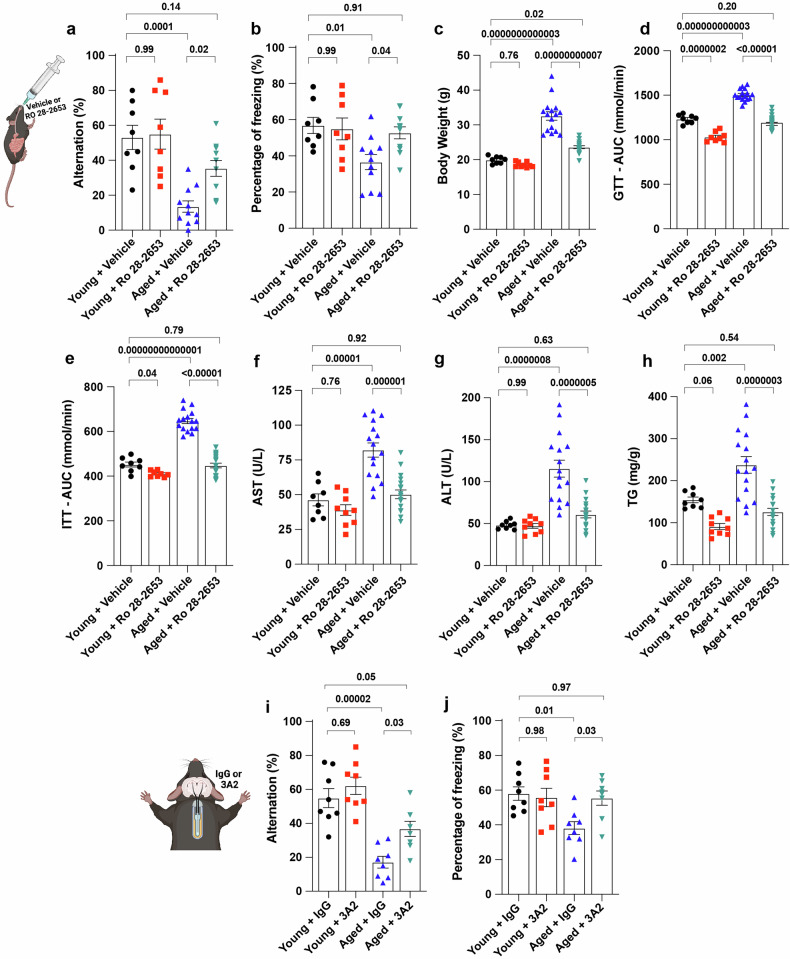
Pharmacological inhibition of MT1-MMP improves memory function and metabolism in 18-month-old aged mice. **a**, **b** Cognitive function assessment using the Y-maze task (**a**) and contextual memory assessment (**b**) by examining the percentage of time freezing. One-way ANOVA followed by Tukey’s multiple comparisons test (*n* = 8 young + vehicle, *n* = 8 young + Ro 28-2653*, n* = 11 aged + vehicle, *n* = 11 aged + Ro 28-2653 mice). **c** Body weight changes at the end of the experiment. One-way ANOVA followed by Tukey’s multiple comparisons test (*n* = 8 young + vehicle, *n* = 9 young + Ro 28-2653*, n* = 16 aged + vehicle, *n* = 18 aged + Ro 28-2653 mice). **d**, **e** The area under curve (AUC) was calculated based on the glucose level of each time point during GTT (**d**) and ITT (**e**). One-way ANOVA followed by Fisher’s LSD post hoc test (*n* = 8 young + vehicle, *n* = 8 young + Ro 28-2653*, n* = 16 aged + vehicle, *n* = 18 aged + Ro 28-2653 mice). **f**–**h** Liver toxicity assessment by examining the serum for AST (**f**), ALT (**g**), and hepatic TG (**h**) levels. One-way ANOVA followed by Tukey’s multiple comparisons test (*n* = 8 young + vehicle, *n* = 9 young + Ro 28-2653*, n* = 16 aged + vehicle, *n* = 18 aged + Ro 28-2653 mice). For 4 weeks, either IgG or 3A2 was administered intrahippocampally. **i**, **j** Following this treatment, cognitive function was assessed using the Y-maze test (**i**) and the contextual fear conditioning test (**j**). One-way ANOVA followed by Tukey’s multiple comparisons test (*n* = 7–8 mice per group). Data are expressed as mean ± SEM, and each data point represents individual mouse.

To substantiate the translational relevance of our observations obtained from studies of genetic loss of function on cognitive function, we orally administrated a MT1-MMP inhibitor (Ro 28-2653) to 20-month-old aged mice and studied their ageing pathologies. Ro 28-2653 is an orally available, brain-penetrant inhibitor with high specificity for MT1-MMP, MMP2, and MMP9. As a hydroxamic acid-based MMP inhibitor, it may also display lower binding affinity for other MMPs, as well as for ADAM and A Disintegrin and Metalloproteinase with Thrombospondin Motifs (ADAMTS) metalloproteinases^31–35^. Compared with 4-month-old control mice group, mice receiving Ro 28-2653 at the dosage of 50 mg/kg/2 days from 20 months to 21 months of age exhibited improved spatial memory in the forced-alternation Y-maze test (Fig. 4a) and enhanced associate memory in the contextual-fear-conditioning test (Fig. 4b). Given the fact that the age-related decline in spatial memory is not regulated by MMP2 and MMP9 (Supplementary Fig. S7b, c), the beneficial effect on memory function conferred by Ro 28-2653 is mainly attributed to its inhibitory effects on MT1-MMP. In line with our previous studies with *Mmp14*^*+/−*^ mice^15,17^, we found that systemic inhibition of MT1-MMP also improved metabolic functions in aged mice. Age-related detrimental changes in body weight and lean mass were all ameliorated in aged mice treated with Ro 28-2653 (Fig. 4c). Impaired glucose metabolism in aged mice, as assessed by glucose tolerance test (GTT) and insulin tolerance test (ITT), was similarly mitigated by the treatment of Ro 28-2653 (Fig. 4d, e). The increases in serum aspartate aminotransferase (AST), Alanine Aminotransferase (ALT), and liver triglycerides (TG) observed in aged mice were suppressed by the treatment of Ro 28-2653 (Fig. 4f–h), indicative of improved liver functions resulting from MT1-MMP inhibition. Notably, Ro 28-2653 exhibited an excellent safety profile in both young and aged mice according to the assessment of body weight and plasma levels of AST and ALT (Fig. 4c, f, g). Taken together, these results demonstrated that blocking MT1-MMP systemically with the brain-penetrant inhibitor at our dose regium phenocopied the improvements in memory function and metabolism observed in aged *Mmp14*^*+/−*^ mice.

Similar observations were obtained in the obese mouse model when subjected to two weeks of Ro 28-2653 (50 mg/kg/day) treatment (Supplementary Fig. S8a). Through the object location memory task, the Y-maze test, and the water maze test, we found that treatment with Ro 28-2653 improved spatial memory and cognition in mice with HFD-induced obesity (Supplementary Fig. S8b–e). Consistent with our previous studies with *Mmp14*^*+/−*^ mice^15,17^, systemic inhibition of MT1-MMP also improved metabolic functions in obese mice as evidenced by the reductions in body weight, blood glucose, TG, and total cholesterol (Supplementary Fig. S8f–i).

To further assess the tissue-specific effect of MT1-MMP inhibition on cognitive function, 18-month-old aged mice were implanted with osmotic pumps that continuously infused a blocking antibody against MT1-MMP (3A2) or an IgG control antibody directly into the hippocampus for one month. The specificity of this blocking antibody has been well-characterized in our previous studies, in which we showed that blocking the activity of MT1-MMP with this antibody effectively promotes body weight loss in obesity, improves metabolic functions in diabetes, and promotes viral clearance in SARS-CoV-2-infected mice^13,15–17^. We then assessed hippocampal-dependent learning and memory performance in the mice. Surprisingly, 3A2-treated mice exhibited improved spatial memory in the forced-alternation Y-maze test (Fig. 4i) and improved associative memory in the contextual fear conditioning test (Fig. 4j). To assess the contribution of hippocampal MT1-MMP to cognitive decline in obesity, we performed the behavior tests in HFD-induced obese mice infused with IgG or 3A2 directly into the hippocampus via an osmotic pump (Supplementary Fig. S8a). Remarkably, blocking MT1-MMP specifically in the hippocampus phenocopied the learning and memory improvements seen in *Mmp14*^*+/−*^ mice with obesity (Supplementary Fig. S8j–m), suggesting that hippocampal MT1-MMP negatively regulates cognitive function in both aging and obesity, and this effect can be reversed by pharmacological MT1-MMP inhibition.

### MT1-MMP activation drives cognitive aging

**Fig. 5:**
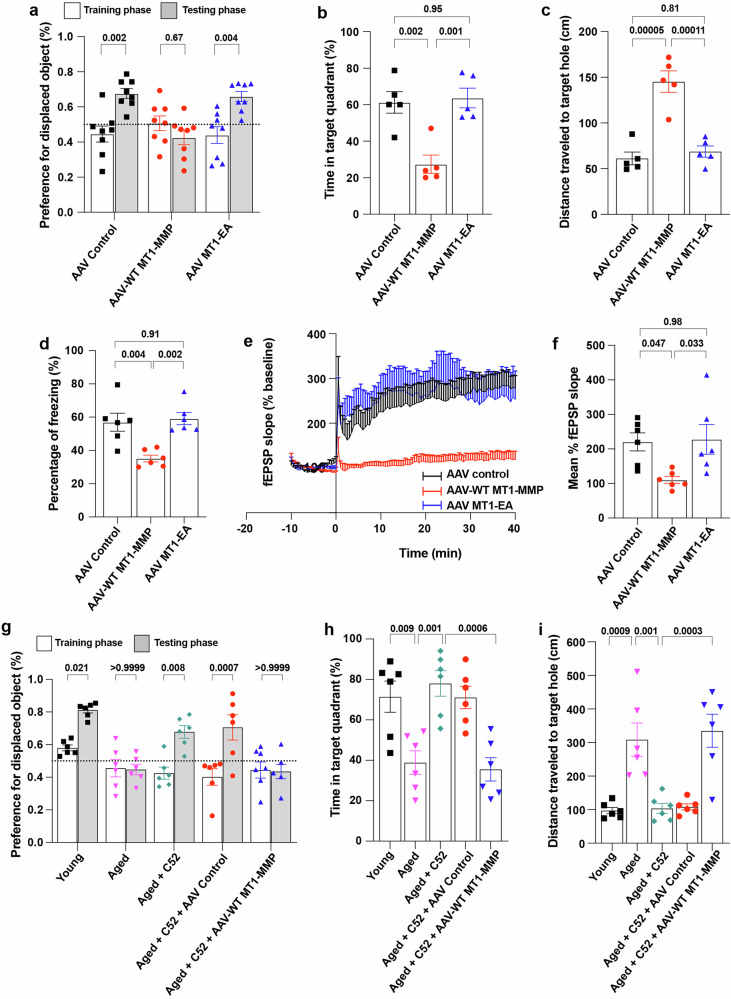
Ectopic expression of MT1-MMP in the hippocampus induces cognitive deficit in 2-month-old young mice. **a** Percentage preference for the displaced object in the object location task from mice receiving either AAV-control, AAV-WT MT1-MMP, or AAV-MT1 EA. One-way ANOVA followed by Tukey’s multiple comparisons test (*n* = 8 mice per group). **b**, **c** Percentage of total time spent in the target quadrant (**b**) and distance traveled to the target hole (**c**) in the Barnes maze test. One-way ANOVA followed by Tukey’s multiple comparisons test (*n* = 5 mice per group). **d** Percentage of freezing time during a contextual fear conditioning test. One-way ANOVA followed by Tukey’s multiple comparisons test (*n* = 6 mice per group). **e**, **f** Changes in fEPSP in the hippocampal CA1 for LTP analysis (**e**) and the average of fEPSP (**f**). One-way ANOVA followed by Tukey’s multiple comparisons test (*n* = 6 mice per group). **g**–**i** Young and aged mice treated with C52 (10 mg/kg/day) for two weeks after AAV injection and tested for spatial memory using object location task (**g**) and Barnes maze test (**h**, **i**). One-way ANOVA followed by Tukey’s multiple comparisons test (*n* = 6 mice per group). Data are expressed as mean ± SEM, and each data point represents individual mouse.

To determine whether an increase in hippocampal MT1-MMP in young mice may exert negative effects on cognitive function similar to the effects of the age-related increase in MT1-MMP on the aged brain, we used an adeno-associated virus (AAV) vector to ectopically express WT MT1-MMP, a catalytically inactive MT1-MMP mutant (MT1 EA) or GFP as a control in the hippocampus of 2-month-old young mice (Supplementary Fig. S9a). Confocal imaging revealed the successful transduction of AAV labeled with mCherry into the hippocampal regions (Supplementary Fig. S9b), as validated by the increased expression of hippocampal MT1-MMP (Supplementary Fig. S9c). Through the object location (Fig. 5a) and the Barnes maze memory tasks (Fig. 5b, c), we found that the ectopic expression of WT MT1-MMP, but not the catalytically inactive MT1-MMP mutant, impaired hippocampus-dependent spatial memory in the young mice. Similarly, contextual memory was reduced in mice transduced with AAV-WT MT1-MMP (Fig. 5d). Consistently, LTP, as measured by electrophysiological recordings, was robustly decreased in mice transduced with AAV-WT MT1-MMP (Fig. 5e, f). No differences in neuroinflammatory markers (Supplementary Fig. S9d) and microglia activation (Supplementary Fig. S9e–g) were detected in the hippocampus of mice transduced with AAV-WT MT1-MMP. These data showed that increased the activation of hippocampal MT1-MMP alone induced deficits in memory and learning in young mice without the involvement of other risk factors associated with cognitive impairment.

To investigate whether this MT1-MMP-induced cognitive impairment was mediated through neuroinflammation, we administered the neuroinflammation suppressor C52 over two weeks in 18-month-old aged mice, which led to significant improvements in several functional outcomes. These beneficial effects of C52 on cognitive function were abrogated by the overexpression of WT MT1-MMP (Fig. 5g–i), suggesting that MT1-MMP drives age-related cognitive impairment independent of neuroinflammation.

### MT1-MMP cleaves GPR158 to inhibit OCN signaling

**Fig. 6:**
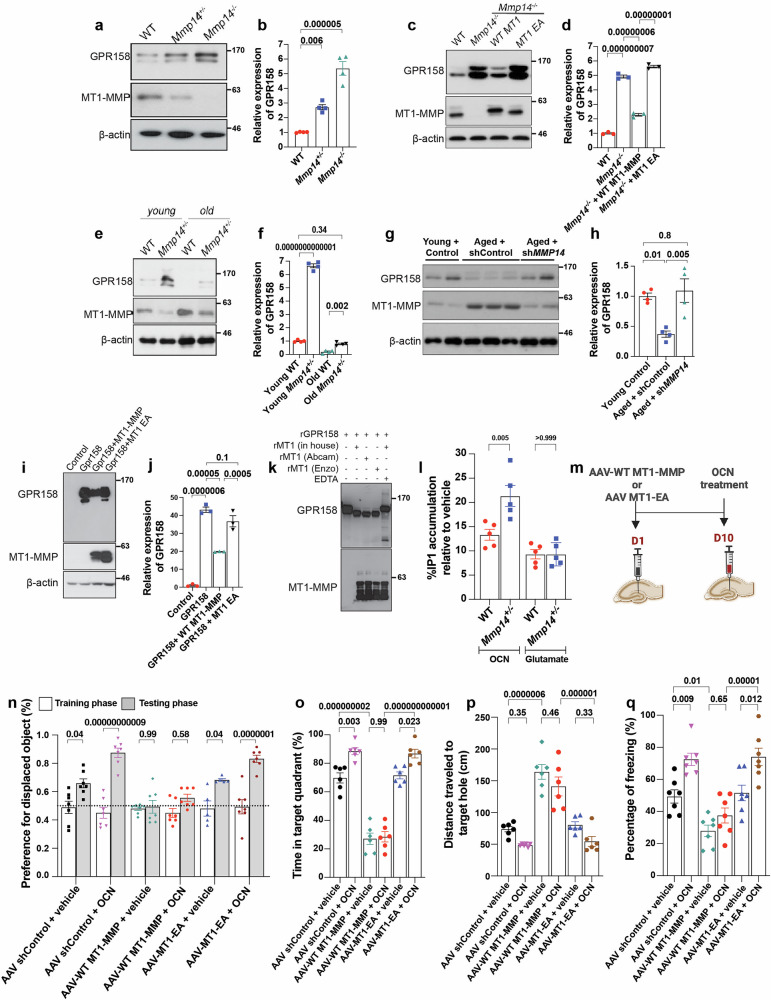
MT1-MMP cleaves GPR158 to suppress OCN signaling. **a**, **b** Western blotting analysis on the expression of GPR158 in the hippocampus lysates from WT, *Mmp14*^*−/−*^ and *Mmp14*^*+/−*^ mice (**a**). Quantification of GPR158 expression relative to β-actin loading control (**b**). One-way ANOVA followed by Tukey’s multiple comparisons test (*n* = 4 mice per group). **c**, **d** The protein expression of GPR158 in primary hippocampal neurons obtained from WT and *Mmp14*^*−/−*^ mice and further treated with either WT MT1-MMP or catalytic inactive MT1-EA (**c**). Quantification of GPR158 expression relative to β-actin loading control (**d**). One-way ANOVA followed by Tukey’s multiple comparisons test (*n* = 3 mice per group). **e**, **f** Western blot analyses of GPR158 and MT1-MMP in hippocampus lysates from young and aged WT and *Mmp14*^*+/−*^ mice (**e**) and quantification (**f**). One-way ANOVA followed by Tukey’s multiple comparisons test (*n* = 4 mice per group). **g**, **h** Representative immunoblots comparing hippocampal GPR158 protein levels in young and aged mice injected with shRNA targeting *Mmp14* (sh*MMP14)* or scrambled shRNA (shControl) (**g**) and quantification of GPR158 expression (**h**). One-way ANOVA followed by Tukey’s multiple comparisons test (*n* = 4 mice per group). **i**–**l** In vitro cleavage assay in HEK293 cells expressing human GPR158. Western blot analyses of GPR158 and MT1-MMP in cell lysates transfected with either WT MT1-MMP or catalytic inactive MT1-EA (**i**). Quantification of the immunoblot for GPR158 expression (**j**). One-way ANOVA followed by Tukey’s multiple comparisons test (*n* = 3 mice per group). rGPR158 was incubated with rMT1 from three different sources (in-house, Abcam, and Enzo) and examined for GPR158 and MT1-MMP expression using western blotting assay (**k**). IP3 accumulation in WT and *Mmp14*^*+/−*^ mice hippocampal neurons after 1 h treatment with OCN or glutamate (**l**). One-way ANOVA followed by Tukey’s multiple comparisons test (*n* = 5 mice per group). **m** Schematic depicting bilateral injection of AAV expressing either AAV-WT MT1-MMP, or AAV-MT1-EA followed by OCN injection after two weeks. **n** The object location task to examine the preference for the displaced object. One-way ANOVA followed by Tukey’s multiple comparisons test (*n* = 7 for AAV shControl + vehicle, AAV shControl + OCN, AAV-WT MT1-MMP + vehicle, AAV-WT MT1-MMP + OCN, AAV-MT1-EA + OCN and *n* = 5 mice for AAV MT1-EA + vehicle). **o**–**q** Percentage of total time spent in the target quadrant (**o**) and distance traveled to the target hole (**p**) in the Barnes maze test. One-way ANOVA followed by Tukey’s multiple comparisons test (*n* = 6 mice per group). Percentage of freezing time during a contextual fear conditioning test (**q**). One-way ANOVA followed by Tukey’s multiple comparisons test (*n* = 7 mice per group). Data are expressed as mean ± SEM, and each data point represents individual mouse.

OCN, a bone-derived hormone whose expression decreases with age, is important for the maintenance of cognitive integrity in aging^36,37^, and its cognition-regulatory functions are mediated by GPR158^37^, an orphan G protein-coupled receptor specifically expressed in neurons of the CA3 region of the hippocampus. Loss of either OCN or GPR158 leads to deficits in hippocampus-dependent memory in mice^37–39^. Considering the importance of OCN/GPR158 signaling in the maintenance of cognitive function, we investigated OCN/GPR158 signaling as a possible intermediary of the detrimental effects of MT1-MMP on age-related cognitive function. We found that the protein expression of GPR158 in the hippocampus was significantly elevated in *Mmp14*^*−/−*^ mice at P15, but the transcriptional level of *Gpr158* was not altered by the loss of MT1-MMP (Fig. 6a, b; Supplementary Fig. S10a), revealing that MT1-MMP regulates GPR158 at the protein level. Consistently, MT1-MMP deficiency increased GPR158 protein levels in primary hippocampal neurons, and the reintroduction of WT MT1-MMP, but not MT1-EA, into *Mmp14*^*−/−*^ neurons restored the expression of GPR158 to WT levels (Fig. 6c, d), suggesting that MT1-MMP regulates GPR158 expression via proteolytic cleavage. Interestingly, reduced GPR158 protein expression in the hippocampus was observed in 18-month-old aged mice (Fig. 6e, f). Haploinsufficiency of MT1-MMP in 18-month-old aged mice restored GPR158 expression to a level comparable to that observed in 2-month-old young mice (Fig. 6f). Consistently, similar results were observed in 18-month-old aged mice with the targeted knockdown of hippocampal MT1-MMP (Fig. 6g, h), revealing that age-related activation of MT1-MMP leads to reduced expression of GPR158 in the hippocampus and that blocking MT1-MMP activity can reverse this change. In line with these observations, we also found reduced expression of GPR158 in the hippocampus of mice with HFD-induced obesity, and this change was reversed by the targeted depletion of hippocampal MT1-MMP (Supplementary Fig. S11a, b). To understand how MT1-MMP cleaves GPR158, HEK293T cells expressing human GPR158 were transfected with either WT MT1-MMP or MT1 EA. A significant reduction in the expression of GPR158 was observed only in cells expressing WT MT1-MMP (Fig. 6i, j), suggesting that the enzymatic activity of MT1-MMP is essential for GPR158 downregulation. To investigate whether MT1-MMP directly cleaves GPR158, recombinant human GPR158 (rGPR158) was incubated with the recombinant catalytic domain of MT1-MMP (rMT1) from three different sources in vitro. The expression of full-length GPR158 was reduced concomitantly with the formation of a truncated form of GPR158 in the presence of rMT1. This cleavage event was inhibited by the potent MT1-MMP inhibitor EDTA (Fig. 6k). The truncated fragment of GPR158 observed in the in vitro assay could not be detected in the cell-based assay, suggesting that it may be subjected to rapid intracellular degradation. These results indicate that MT1-MMP proteolytically and directly cleaves GPR158.

### MT1-MMP restrains the ability of OCN/GPR158 signaling to promote cognition

To assess the functional implications of GPR158 cleavage on OCN signaling, we isolated hippocampal neurons from WT or *Mmp14*^*−/−*^ mice and stimulated them with OCN. We found that the loss of MT1-MMP enhanced the OCN-induced accumulation of inositol 1,4,5-trisphosphate (IP3), a second messenger used by GPR158, in primary hippocampal neurons (Fig. 6l). In contrast, glutamate-induced IP3 accumulation in neurons did not differ between *Mmp14*^*−/−*^ and WT mice (Fig. 6l), suggesting that MT1-MMP specifically regulates OCN/GPR158 signaling in hippocampal neurons. Next, we injected AAV expressing either AAV-WT MT1-MMP or catalytically inactive AAV-MT1-EA into the dorsal hippocampus of 3-month-old WT mice. Two weeks later, OCN was injected at the same stereotaxic coordinates (Fig. 6m). The local delivery of OCN improved hippocampus-dependent memory in both the control mice and mice injected with AAV-MT1-EA as observed in the novel object location task (Fig. 6n) and Barnes maze task (Fig. 6o–q). These effects were abrogated in mice with ectopic AAV-WT MT1-MMP expression (Fig. 6n–p). Similarly, ectopic expression of hippocampal MT1-MMP abolished the ability of OCN to improve contextual memory (Fig. 6q). These results suggest that MT1-MMP counteracts the beneficial effect of OCN on memory function. Moreover, the plasma level of OCN was unaltered in aged *Mmp14*^*+/−*^ mice **(**Supplementary Fig. S10b), suggesting that the ability of Mmp14 haploinsufficiency to promote cognition is independent of OCN bioavailability. Together, these results demonstrate that MT1-MMP, which is activated with aging, cleaves GPR158 to suppress the ability of OCN to promote cognition, thereby leading to a decline in memory function and learning capacity during aging.

### MT1-MMP depletion restores the cognition-promoting effect of OCN in obese mice

We next investigated whether MT1-MMP may similarly restrain the cognition-promoting effect of OCN in the context of obesity. 2-month-old WT or *Mmp14*^*+/−*^ mice were fed with HFD for 12 weeks and then treated with the systemic administration of recombinant OCN. Surprisingly, treatment with OCN at our dose regime failed to improve memory functions in obese mice, as assessed by the object location memory task, the Y-maze test, and the water maze test (Supplementary Fig. S12a–d). Similarly, exogenous OCN also did not restore synaptic plasticity in obese mice (Supplementary Fig. S12e), indicating that obesity may be a state of resistance for OCN in term of cognitive function. In contrast, haplodeficiency in MT1-MMP improved memory and synaptic plasticity in obese mice (Supplementary Fig. S12a–e). All such effects were further potentiated in *Mmp14*^*+/−*^ mice treated with OCN (Supplementary Fig. S12a–e). These results demonstrate that obesity-indued activation of MT1-MMP suppresses the ability of OCN/GPR158 signaling to promote cognition and that MT1-MMP depletion sensitizes obese mice to the cognition-promoting effect of OCN.

### MT1-MMP controls cognitive integrity by regulating OCN/Gpr158 signaling

**Fig. 7:**
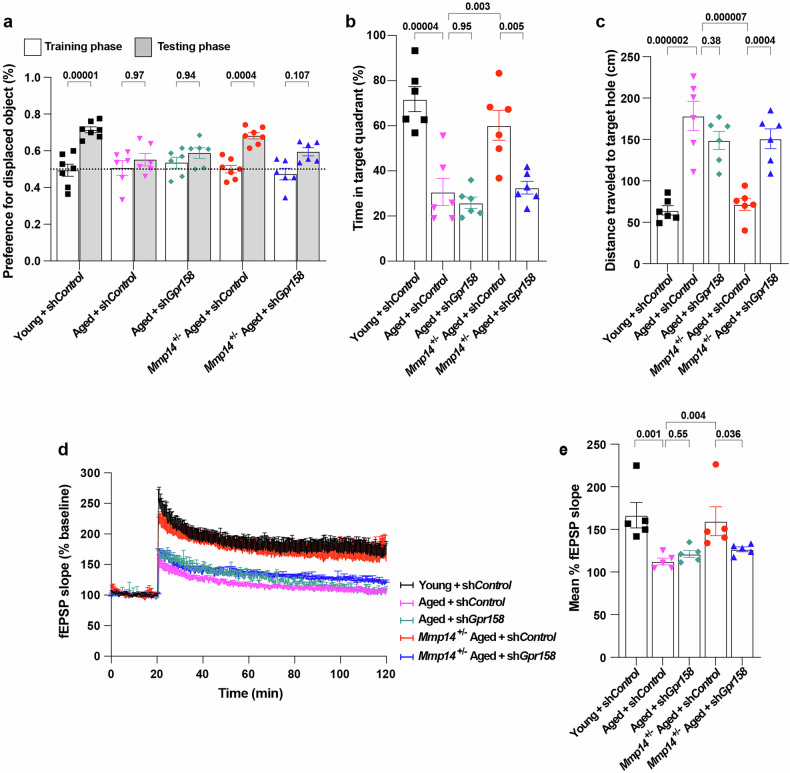
MT1-MMP regulates cognitive aging by suppressing Ocn/Gpr158 signaling axis. **a** The preference for the displaced object in the object location task by 2-month-old (young), 18-month-old (aged) WT and *Mmp14*^*+/−*^ aged mice injected with either shControl or sh*Gpr158*. One-way ANOVA followed by Tukey’s multiple comparisons test (*n* = 7 young + shControl, *n* = 6 aged + shControl, *n* = 6 aged + sh*Gpr158, n* = 7 *Mmp14*^*+/−*^ aged + shControl, *n* = 6 *Mmp14*^*+/−*^ aged + sh*Gpr158)*. **b**, **c** Percentage of total time spent in the target quadrant (**b**) and distance traveled to the target hole (**c**) in Barnes maze test. One-way ANOVA followed by Tukey’s multiple comparisons test (*n* = 6 mice per group). **d**, **e** LTP analysis in the CA1 hippocampus region by measuring the fEPSP (**d**) and the average of fEPSP slope (**e**). One-way ANOVA followed by Fisher’s LSD post hoc test (*n* = 5 mice per group). Data are expressed as mean ± SEM, and each data point represents individual mouse.

To investigate whether increased OCN-GPR158 signaling is responsible for the restoration of memory function in aged *Mmp14*^*+/−*^ mice, we generated 18-month-old *Mmp14*^*+/−*^*Gpr158*^*hippo+*^ mice in which *Gpr158* was specifically knocked down in the hippocampus via a lentiviral vector. In accordance with our previous findings (Fig. 2), aged *Mmp14*^*+/−*^ mice presented significant improvements in memory functions (Fig. 7a–c) and hippocampal plasticity (Fig. 7d, e), the levels of which decreased with age in both the sh-*control* and sh-*Gpr158*-treated WT mice. The targeted depletion of *Gpr158* in the hippocampus abolished the beneficial effects of *Mmp14* heterozygosity on memory functions and synaptic plasticity (Fig. 7a–e). These findings suggest that MT1-MMP regulates age-related memory functions in a GPR158-dependent manner.

## Discussion

Aging is accompanied by progressive alterations in the brain’s ECM composition and cellular microenvironment, which collectively contribute to cognitive decline^40^ and increased susceptibility to neurodegenerative diseases such as Alzheimer’s disease^41^.

Our study, for the first time, reveals that the proteolytic event mediated by MT1-MMP has a unique involvement in maintaining cognitive integrity in aging and obesity. Our findings suggest that cognitive decline associated with aging/metabolic disorders can be reversed by inhibiting MT1-MMP. First, we revealed a fundamental vulnerability of the brain, in which activation of MT1-MMP alone can trigger memory decline and hippocampal plasticity deficits, independent of neuroinflammation and blood-brain barrier disruption. Cognitive integrity is specifically regulated by MT1-MMP, but not by other MMPs that have been implicated in pathological neurodegenerative diseases. These observations reveal a new non-canonical regulation of cognitive functions in aging/metabolic disorders. Second, blocking MT1-MMP can restore hippocampal function and plasticity in aged mice, maintaining homeostasis seen in young mice. Similarly, depleting hippocampal MT1-MMP rescues cognitive deficits in diet-induced obese mice. These results, for the first time, reveal that cognitive impairment in aging and obesity is driven by a common mechanism in which MT1-MMP-mediated control of OCN/GPR158 signaling plays a dominant role in mediating memory functions and synaptic plasticity.

The dysregulation of MMP activity has been implicated in various pathological conditions, including cognitive decline and neurodegenerative diseases^11,12^. Targeting MMP activity has emerged as a potential therapeutic approach for treating cognitive impairment and neurodegenerative diseases. In preclinical studies, MMP inhibitors have shown promise in preserving synaptic function and improving cognitive performance in models of pathological neuropathies, such as Alzheimer’s disease and traumatic brain injury^18,19^. However, the clinical translation of MMP inhibitors has faced challenges due to the complex and context-dependent roles of MMPs in different tissues and at various stages of disease progression^42^. Understanding the specific roles of MMPs in distinct disease contexts and developing targeted therapeutic strategies may hold promise for mitigating cognitive impairment associated with neurodegenerative diseases, obesity, and other conditions.

MT1-MMP expression is markedly upregulated with age in the hippocampus of 5xFAD mice model of Alzheimer’s disease. Notably, MT1-MMP levels are significantly elevated in hippocampal fractions by 6 months of age, coinciding with pronounced cognitive deficits, including impairments in spatial learning and memory, as well as synaptic dysfunction^43^. Similarly, elevated expression of MMP-14 has been detected in the brains of individuals with Alzheimer’s disease. This dynamic upregulation of MMP-14 in both murine models and human disease highlights its potential role as a mediator of neurodegeneration and cognitive impairment under both physiological and pathological conditions.

In this study, we demonstrated that MT1-MMP blockade can rejuvenate the aging brain by restoring hippocampal function and plasticity, independent of other risk factors associated with cognitive aging. Additionally, our findings indicate that MT1-MMP plays a crucial role in regulating cognitive functions in the context of obesity. In contrast, genetic ablation of either MMP2 or MMP9, two major downstream targets of MT1-MMP linked to neurodegenerative diseases, did not alter memory functions in aged mice. These results suggest that MT1-MMP may play an indispensable role in maintaining cognitive integrity in both aging and obesity.

Dysregulation of the OCN/GPR158 pathway is a hallmark of age-related memory loss. The underlying molecular mechanism of this dysregulation remains largely unknown. We are the first to report MT1-MMP as a regulator of GPR158, revealing that MT1-MMP-mediated proteolytic cleavage of GPR158 is crucial for OCN-dependent cognitive functions in aging and obesity. We also for the first demonstrated that OCN/GPR158 signaling is vital for cognitive integrity in metabolic dysfunctions and that obesity induces resistance to OCN’s cognition-promoting effects via MT1-MMP-mediated cleavage of GPR158. Our insights into OCN/GPR158 signaling regulation suggest a physiological mechanism for endogenous OCN sensitivity in the central nervous system in aging and obesity.

Aging results in a decrease in OCN bioavailability and an MT1-MMP-mediated reduction in GPR158 expression, both of which contribute to impaired OCN/GPR158 signaling and consequently, age-related cognitive decline. These observations are further supported by genetic evidence showing that mice with partial deficiencies in both OCN and GPR158 (*Ocn*^+/*−*^*Gpr158*^+/*−*^) exhibit age-related cognitive impairment, whereas mice with a partial deficiency in either *Ocn* (*Ocn*^+/*−*^) or *Gpr158* (*Gpr158*^+/*−*^) alone do not display such cognitive deficits^37–39^.

The proteolytic activity of MT1-MMP was physiologically inhibited by tissue inhibitor of metalloproteinase-2 (TIMP-2). Previous studies demonstrated that human umbilical cord plasma, enriched in TIMP2, significantly improved behavioral performance, LTP, and neuronal activation in aged mice^44^. Administration of recombinant TIMP2 to aged WT mice recapitulated these hippocampus-dependent cognitive benefits. More recently, TIMP2 has been reported to exert MMP-independent effects, resulting in enhanced cognition and increased synapse density in aged mice. Beyond its classical role as an MMP inhibitor, TIMP2 has been shown to participate in diverse protein-protein interactions, including those with α3β1-integrin^45^, low-density lipoprotein receptor-related protein^46^, insulin-like growth factor-1 receptor, and vascular endothelial growth factor-A^47^. Many of these binding partners have established roles in synaptic plasticity^48–51^. Thus, it remains possible that the cognitive improvements observed following peripheral TIMP2 administration in aged mice are mediated through interactions with these additional binding partners, or others yet to be identified. Therefore, further investigation is required to conclusively determine the precise mechanisms by which TIMP2 enhances cognitive function during aging.

We report the identification of a direct negative regulator of GPR158, establishing MT1-MMP as a potential therapeutic target for preserving cognitive integrity in aging and obesity. Although further investigations are necessary to validate the safety of MT1-MMP inhibition, preliminary evidence suggests that MT1-MMP inhibition would not result in developmental defects under physiological conditions, as *Mmp14* heterozygous mice do not exhibit any gross phenotypes during aging. This indicates that MT1-MMP inhibition may specifically target the pathophysiology of aging and metabolic disorders without affecting normal development. In fact, our previous studies, along with the current findings, demonstrate that genetic ablation or pharmacological inhibition of MT1-MMP confers protection against multiple age-associated disorders, including cognitive decline, obesity, and diabetes, without adverse effects^15–17^. These observations reduce safety concerns in drug development. Taken together, these findings reveal an opportunity to treat multiple age-related diseases, including neurodegenerative disorders, obesity, diabetes, arthritis, and atherosclerosis, with a single MT1-MMP-blocking agent.

## Materials and methods

### Animals

This study was conducted in accordance with the guidelines of the Committee on the Use of Human & Animal Subjects in Teaching and Research at Hong Kong Baptist University. All the procedures were approved by the Department of Health under Hong Kong legislation. All mice were housed in an environmentally controlled, pathogen-free barrier facility on a 12-h light-dark cycle with constant ambient temperature (22–24 °C) and humidity (~60%), food and water available ad libitum. C57BL/6 J male mice used for the experiment were bred in our laboratory using mice purchased from The Jackson Lab. *Mmp14*^*+/−*^ mice have been previously described in our published studies^15,17^. All behavior experiments were conducted in the animal facilities of Hong Kong Baptist University and the University of Hong Kong.

### HFD induced obesity model

Following weaning, mice were housed individually under standard laboratory conditions and assigned to receive either an HFD (60% kcal from fat, Research Diets D12492) or a CD (10% kcal from fat, Research Diets D12450J). In mice subjected to HFD feeding, intraperitoneal injection of 1 μg/kg rOCN (YP002682MO, Cusabio) was performed for 1 week, followed by neurobehavioral assessments. Mice were maintained on their respective diets and specific experimental treatments for the durations specified in each result.

### C52 administration

C52 (Sigma-Aldrich) was dissolved in a solution containing 40% polyethylene glycol (Sigma-Aldrich) and 60% of a 30% Kolliphor HS15 solution (Sigma-Aldrich). Both aged mice (18 months old) and young mice (2 months old) received C52 via oral gavage at a dose of 10 mg/kg once daily for 30 consecutive days. Neurobehavioral assessments were conducted two weeks after the initiation of dosing. Following completion of the 30-day treatment period, mice were euthanized and hippocampal tissues were rapidly harvested for subsequent gene expression analysis.

### Ro 28-2653 treatment

The selective MMP inhibitor Ro 28-2653 (AOB2296, Aobious) was orally administered every other day at a dosage of 50 mg/kg. For the aged mice group, treatment was initiated at 20 months of age and continued for 4 weeks until the mice reached 21 months. In the young mice group, treatment began at 2 months of age and continued for 4 weeks. Age-matched control groups received equivalent volumes of vehicle control saline containing 2.0 mg/mL sodium carboxymethyl cellulose, 0.9 mg/mL propyl paraben, and 0.1 mg/mL methyl paraben^34^. Neurobehavioral assessments were performed seven days prior to the final drug administration.

### Neurobehavioral assessment

#### Novel object location test

The object location memory task was performed as described previously^52^. Mice were placed in an open box (50 *×* 50 *×* 50 cm) to acclimate to the testing environment for five min (habituation phase). This was followed by a training session where two different objects were placed in predetermined locations within the open box, and the mice were allowed to freely explore the objects for 10 min. Three consecutive trials were conducted with intertrial intervals of 3 min during the training session. After each training session, both objects were wiped with 70% ethanol to remove any residual odor clues. After 24 h, mice were reintroduced to the open box for 10 min, but one of the familiar objects was displaced to a new location. Exploration time for each object during the training and testing sessions was recorded using ANY-maze tracking software. Mice that accumulated less than 10 s of total exploration time across both objects during the training session were excluded from subsequent analyses^53^.

#### Barnes Maze test

A four-day experiment was conducted as described previously with slight modifications^54^. The Barnes maze consists of a white circular platform 90 cm in diameter and elevated 70 cm above the ground. Twenty holes were evenly spaced around the perimeter of the platform, with one hole leading to an escape box. The position of the escape box was kept the same throughout the experiment to provide a consistent target location for the mice. Visual cues were placed around the maze to help mice navigate and locate the escape box. During the habituation (day 1) and training phase (days 2–3), mice were placed in the center of the platform, and the bright lights were turned on. Mice were allowed to explore freely for 3 min to locate the escape box and stay there for 2 min with the lights off. If the mice failed to find the escape box, they were guided to it and allowed to stay there for 2 min. The training phase consisted of five trials over two days to allow the mice to learn the location of the escape box. A probe trial was conducted on day four to assess the memory retention ability of the mice. During the 3-min probe trial, the escape box was removed, and mice were placed on the platform with bright lights until they entered the target hole. Mice that failed to leave the center of the platform within 60 s or that remained immobile and did not explore the maze were excluded from analysis^55^. All the mice’s movements were recorded and tracked using an overhead camera connected to the ANY-maze tracking software. Data were analyzed for the distance traveled to the target hole and the time spent in the target quadrant during the probe trial.

#### Contextual fear conditioning test

The fear conditioning test was performed as described previously with slight modification^56^. The conditioning chamber contains a grid of metal rods that deliver mild foot shocks as an unconditioned stimulus (US). The chamber had overhead lighting for visual cues and an audio speaker for conditioned stimuli (CS), such as white noise. On training day 1, mice were exposed to white noise at 55 dB as a CS for 30 s, followed by a 0.8 mA foot shock as the US during the last 2 s. Each mouse was exposed to three CS-US repeats during the 5-min training period. After 24 h, mice were tested for contextual fear memory in the same testing chamber by measuring freezing behavior. The mice were allowed to freely explore the chamber for 5 min without foot shocks or other aversive stimuli. All movements were recorded using an overhead camera. Immobility for more than 2 s was considered freezing and was automatically analyzed using ANY-maze tracking software. Mice showing persistent immobility or unusually high freezing behavior during training were excluded from final analyses.

#### Open field test

The open field test was performed in a Plexiglas square box (50 *×* 50 *×* 50 cm) with an overhead camera. Mice were placed in the middle of the box and allowed to move freely for 15 min. All movements were tracked using ANY-maze tracking software. The box was cleaned with 70% ethanol and dried before the next mouse was placed. The total distance traveled during the test was analyzed to examine motor function. Mice that remained immobile for an extended period (e.g., more than 50% of the session) or failed to leave the starting position were excluded from analysis^57^.

#### Wire hang test

Mice were placed on an iron wire mesh suspended 50 cm above the ground, with a bedding layer underneath to prevent injury from falling. The mesh was slightly shaken to encourage the mice to grip the wire, and then it was gently turned upside down to evaluate the latency to fall. Each mouse was recorded for a maximum of 3 min. Mice that failed to grasp the wire mesh at the start of the test after three attempts were excluded from the analysis^58^.

#### Nesting test

The mice’s nest-building capacity was evaluated in their home cage. Each mouse was given one piece of nest material, typically a Nestlet (Ancare, USA) weighing around 3 g, placed on the floor in the center of the cage. After 12 h, the nests were evaluated using a 5-point scoring scale, as described previously. A nesting score of 0 was assigned if the Nestlet had not been moved or no sign of interaction was observed; a score of 1 if interaction with the nesting material was evident but it had not been gathered to a nest site; a score of 2 if the nesting material had been assembled to form a nest site; a score of 3 if the nest had identifiable walls that created a ‘cup’ or ‘bowl’ shape; a score of 4 if an incomplete dome was formed; and a score of 5 for a full dome nest. Mice for which nesting scoring was not possible due to observer error were excluded from the analysis^59^.

#### Gait analysis

Mice were placed on a 60 cm-long platform after inking their paws with a nontoxic red color. The stride length was calculated manually by measuring the distance between the central regions of two consecutive footprints of the same limb (fore/hind)^60^. A minimum of three consecutive footprints was selected to calculate the stride length. Mice displaying refusal to walk, frequent stopping, or excessive grooming or rearing behaviors during the test walk were not included in the final analysis.

#### Y-Maze test

Mice were examined for spontaneous alternation using a Y-shaped maze with arms of equal length (30 cm long and 10 cm wide) interconnected at a 120° angle. Mice were placed in the center and allowed to freely explore all the arms for 8 min. Each mouse was recorded to calculate the sequence of arms entered. Spontaneous alternation was defined as the tendency of a mouse to enter three different arms of the Y-maze in consecutive choices and was calculated using the following formula: Spontaneous alternation percentage = (number of alternations)/(number of arms entered − 2) *×* 100. If a mouse remained immobile or failed to explore all three arms, it was excluded from the study^61^. After each trial, the Y-maze was wiped with 75% ethanol to avoid odor cues.

#### Morris water maze test

Spatial memory and learning were assessed using the Morris water maze as described previously with slight modification^62^. A round tank (150 cm in diameter and 60 cm deep) was filled approximately halfway with opaque water, and the temperature was maintained at 24–25 °C throughout the experiment. The maze was divided into four equal quadrants, and four visual cues were placed on the walls to serve as spatial reference points for each quadrant. Mice were trained for five days with four daily trials, with an intertrial interval of 15 min. Mice could swim freely in the water maze to search for the hidden platform (10 cm in diameter) within 90 s. If a mouse failed to find the hidden platform within the designated time, it was gently guided to the platform and allowed to remain there for 15 s. All movements were tracked with ANY-maze tracking software to measure the distance traveled to reach the platform and the time spent in the target quadrant. Mice exhibiting immobility, passive floating, or a lack of active swimming during the trial sessions were not included in the final analysis^63^.

#### Glucose tolerance test

Mice were tested for glucose tolerance at the end of the study. For the glucose tolerance test, mice were fasted overnight (12 h) in clean cages with no food supplied but with free access to drinking water. Fasted mice were intraperitoneally injected with a 1 mg/kg glucose solution. Blood glucose levels were measured 15, 30, 45, 60, 90, and 120 min after glucose injection by placing a small drop of tail vein blood on OneTouch Ultra glucometer test strips (LifeScan).

#### Insulin tolerance test

Mice were fasted for 6 h and were then intraperitoneally injected with 0.5 mg/kg of insulin. Blood glucose levels were measured from tail blood at 15, 30, 45, 60, 90, and 120 min after the insulin injection.

### Electrophysiology

The experiments were carried out as previously described. Mice were anesthetized using isoflurane inhalation and then euthanized by cervical dislocation. The brain was harvested, ensuring minimal damage to the tissue, and the hippocampus was dissected in ice-cold (4 °C) artificial cerebrospinal fluid (ACSF; Tocris Bioscience). ACSF was saturated with carbogen (95% O_2_/5% CO_2_) to maintain oxygenation. Using a vibratome, the hippocampus was sliced into thin sections of 350 μm nominal thickness. The slices were allowed to incubate with oxygenated ACSF for around 1 to 2 h at room temperature before being used for recording. The hippocampal slices were positioned on a multi-electrode array for recording field excitatory postsynaptic potentials (fEPSPs) in the CA1 region. Stimulation was applied to downstream electrodes in the CA1 and CA3 regions along the Schaffer collateral pathway, and the fEPSP signals were acquired using the MultiClamp 700 A amplifier (Axon Instruments). To induce LTP, three episodes of theta-burst stimulation (TBS) were used. Each TBS consisted of 10 bursts of 4 stimuli at 100 Hz, with an interburst interval of 200 ms. LTP was calculated by the formula: (fEPSP value after TBS − mean baseline fEPSP value)/mean baseline fEPSP value) *×* 100.

### Intrahippocampal injections

For lentiviral gene transfer, the shRNA sequence targeting mouse *Mmp14* and *Gpr158* was cloned into the cPPT-CMV-eGFP-2A-PTN-WPRE lentiviral vector (Addgene). The modified plasmids were co-transfected into HEK293T cells with pVSVG (packaging vector, Addgene) to generate shMmp14, shGpr158, or scramble shRNA (control) expressing lentivirus. AAV-WT MT1-MMP and AAV-MT1-EA virus produced by the AAV8-CaMKIIα:mCherry-WPRE-bGH vector were purchased from Addgene.

Mice were anesthetized via intraperitoneal injection of ketamine/xylazine and secured to a stereotaxic apparatus platform. A midline incision of 10 mm was made to locate the bregma and lambda for precise microinjection. AAV or lentivirus was stereotaxically injected bilaterally into the dentate gyrus (DG) region of the hippocampus using the following coordinates: –1.8 mm anteroposterior (AP), 1.8 mm mediolateral (ML), –1.9 mm dorsal/ventral (DV). The viral titer of 1 × 10^9^ was infused using a Hamilton syringe at a rate of 0.2 μL/min, and the needle was left in place for 5 min to minimize the risk of backflow or leakage.

Intrahippocampal lentiviral injection was performed at 18 months of age, whereas in the young cohort, lentivirus was administered at 2 months of age. Following surgery, animals were monitored daily and housed under standard conditions for recovery. Neurobehavioral assessments were conducted two weeks after lentiviral injection. For AAV experiments, young mice at 2 months of age received hippocampal injections of either AAV encoding AAV-WT MT1-MMP or AAV-MT1-EA. Mouse rOCN (10 ng/μL, YP002682MO, Cusabio) was injected at the exact coordinates two weeks after the AAV injection. Two weeks following AAV administration, neurobehavioral testing was performed.

### Intrahippocampal bilateral cannulation for MT1-MMP inhibitor infusion

For four weeks, IgG or 3A2 was administered intrahippocampally using an osmotic pump. The mice were anesthetized with isoflurane, and their heads were secured in a stereotaxic apparatus. Cannulas (30 G) were implanted in the hippocampus at identical coordinates and fixed in place with dental cement. The cannulas were connected to osmotic pumps (ALZET) implanted subcutaneously in the neck muscle. IgG or 3A2 was continuously released at a rate of 0.25 μL/h over the four-week period. In the aged mice group, treatment commenced at 18 months of age, while in the young mice, pump implantation and antibody infusion began at 3 months of age.

### Primary hippocampal culture

Hippocampi were dissected from mouse embryos (embryonic day 18.5). To dissociate the cells, the hippocampi were digested with 0.05% trypsin and 0.02% EDTA (25300054, Thermo Fisher Scientific) for 15 min at 37 °C. After digestion, the cells were washed three times with DMEM (11995073, Thermo Fisher Scientific) supplemented with 10% FBS (A5670701, Thermo Fisher Scientific), 100 U/mL penicillin-streptomycin (15070063, Thermo Fisher Scientific), and 1× GlutaMAX (35050061, Thermo Fisher Scientific). The tissue was gently triturated using a fire-polished glass pipette to dissociate the cells and then plated. Once the culture was established, the medium was changed twice a week. The culture medium consisted of Neurobasal™ medium (21103049, Thermo Fisher Scientific) supplemented with B-27™ supplement (17504044, Thermo Fisher Scientific) and 1× GlutaMAX. OCN (3 ng/mL) and glutamate (10 ng/mL) were added to the neuronal culture, and IP3 levels were checked after 1 h using an ELISA assay kit (72IP1PEA, Revvity) according to the provided protocol.

### Blood transfusion

Mice were briefly anesthetized, and blood was collected by retro-orbital bleed from young (6–8 weeks) and aged (24 months) mice. Young mice were systematically administered with aged blood (150 μL) or equivalent saline through intravenous tail vein injection for 24 days (8 times).

### ELISA assay

Blood samples were collected in EDTA pre-coated tubes by carefully puncturing the retroorbital sinus using the glass microhematocrit tube. The samples were centrifuged at 1000× *g* for 15 min at 4 °C to collect the plasma. Plasma OCN levels were quantified using the mouse OCN ELISA kit (EEL003, Invitrogen) according to the manufacturer’s protocol.

### Cytokine assay

Hippocampus cytokine levels were analyzed using Mouse XL Cytokine Premixed Kit, Luminex® Performance Assay (FCSTM20, R&D Systems) according to the manufacturer’s instruction.

### RNA extraction and gene expression analysis

Total RNA was extracted from the hippocampus region using TRIZOL reagent (Invitrogen). The extracted RNA was subjected to a genomic DNA elimination step by incubating it for 2 min at 42 °C with a gDNA eraser. The RNA was reverse transcribed to cDNA using the PrimeScript RT Master mix (Takara, RR047) according to the manufacturer’s protocol. Quantitative real-time polymerase chain reaction (qPCR) was performed using TB Green® Premix Ex Taq™ II (Takara, RR820A) on a ABI ViiA 7 Real-time PCR System (Applied Biosystems). The relative fold-change of each gene was calculated using the 2^–ΔΔCt^ method, and *Gapdh* was used for normalization. All the primers used are listed in Supplementary Table S1.

### Western blotting

Western blot was performed as previously described in ref. ^64^. Total protein was extracted from cell or tissue samples using ice-cold RIPA buffer (89901, Thermo Scientific). Samples were homogenized and lysates were centrifuged at 4 °C for 10 min at 14,000× *g* to collect the supernatant. The denatured protein samples (10 μg) were separated by 4%–12% SDS-PAGE using MOPS-SDS running buffer. Proteins were transferred to polyvinylidene difluoride (PVDF) membranes and blocked for 1 h using 5% QuickBlock™ Western Blocking Solution (P0252, Beyotime). Membranes were incubated overnight at 4 °C with primary antibodies, followed by HRP-conjugated secondary antibodies for 1 h at room temperature. Immunoreactive bands were visualized by detecting chemiluminescence using an ECL kit (GE Healthcare). The antibodies used in this study include the following: anti-MT1-MMP antibody (ab51074, Abcam; 1:2000 dilution), anti-Gpr158 (ABIN486340, Antibodies-online; 1:500 dilution), anti-β-actin (HRP conjugate) (5125, Cell Signaling; 1:5000 dilution), and goat anti-rabbit antibody conjugated with HRP (sc-2004, Santa Cruz; 1:2000 dilution).

### Immunohistochemistry

The brain was harvested and post-fixed with 4% paraformaldehyde, followed by overnight cryoprotection in 30% sucrose at 4 °C. Tissue was embedded using Tissue-Tek optimum cutting temperature compound (O.C.T. Sakura Finetek USA), and 35-μm coronal hippocampus sections were obtained. Sections were imaged using a Confocal Laser Scanning Microscope (Leica TCS SP8).

### Examination of BBB permeability

The permeability of the BBB was assessed by quantifying the extravasation of albumin-Evans blue dye into the brain^65^. Evans blue (4 mL/kg) was injected intraperitoneally into the mice one hour before euthanasia. Mice were transcardially perfused with saline, and the brain was quickly removed and homogenized in PBS (pH 7.4). Homogenates were treated with 50% trichloroacetic acid, followed by centrifugation and supernatant collection. The concentrations of albumin-Evans blue in the brain samples were measured using a spectrophotometer at 610 nm. Using a standard curve, the concentration was calculated as μg of albumin-Evans blue dye per mg of brain tissue.

### MMP14 activity assay

MMP14 activity was measured using the Human MMP-14 activity assay kit (QZBMMP14H, Quickzyme Biosciences) according to the manufacturer’s instructions. Samples were added to the anti-MT1-MMP pre-coated microplate and incubated overnight at 4 °C. On day 2, assay buffer 50 μL was added, and after 2 h of incubation at 37 °C, detection reagent (50 μL) was added. Absorbance was recorded at 405 nm every 1 h, and the sample concentration was calculated using the standard curve.

### Transfection and cell line experiments

To investigate the in vitro cleavage of GPR158 by MT1, human embryonic kidney 293 (HEK293) cells were seeded in 6-well plates and transfected with either WT MT1-MMP or a catalytically inactive MT1 EA. Seventy-two hours post-transfection, cells were harvested, and protein expression was analyzed through western blotting. Additionally, rGPR158 proteins (10286-GP, R&D Systems) were incubated with rMT1 (BML-SE259-0010, Enzo Lifesciences), rMT1 (ab157068, Abcam), or in-house produced rMT1 for 16 h, and the resulting mixtures were subjected to western blot analysis.

### Analysis of MMP14 expression and pro-inflammatory cytokines in the human hippocampus using Human Protein Atlas (HPA) and GTEx project

To investigate the expression profile of MMP14 in the human hippocampus, we utilized publicly available RNA-sequencing data from the Genotype-Tissue Expression (GTEx) project, as accessed through the Human Protein Atlas (HPA)^66^. The HPA integrates GTEx transcriptomic data across diverse human tissues. We queried the gene symbol of interest, and the expression data (measured in transcripts per million, nTPM) were extracted from the “Brain - Hippocampus” dataset. To analyze age-associated changes, donor samples were further sorted by age and categorized into two groups: “young” (20–39 years) and “aged” (70–79 years). Pearson’s correlation coefficient analysis was performed between MMP14 and selected pro-inflammatory gene markers using Prism software.

### Statistical analyses

The statistical analyses were performed with GraphPad Prism 8.0 (GraphPad Software, LLC, San Diego, CA). All the data are expressed as mean ± SEM, and the statistical significance was set at *P* < 0.05. The sample size (*n*), post-hoc test, and exact *P* values for each experiment are described in the figure legends.

## Supplementary information

Supplementary Information
