# Supplementary material for: MT1-MMP inhibition rejuvenates ageing brain and rescues cognitive deficits in obesity
Source: Cell Discov. 2025 Sep 23;11:76. doi: 10.1038/s41421-025-00825-w (PMC12454644; doi:10.1038/s41421-025-00825-w)
Supplement: Supplementary file 1 — Supplementary Information [file 41421_2025_825_MOESM1_ESM.pdf]

**Supplementary Fig. S1**

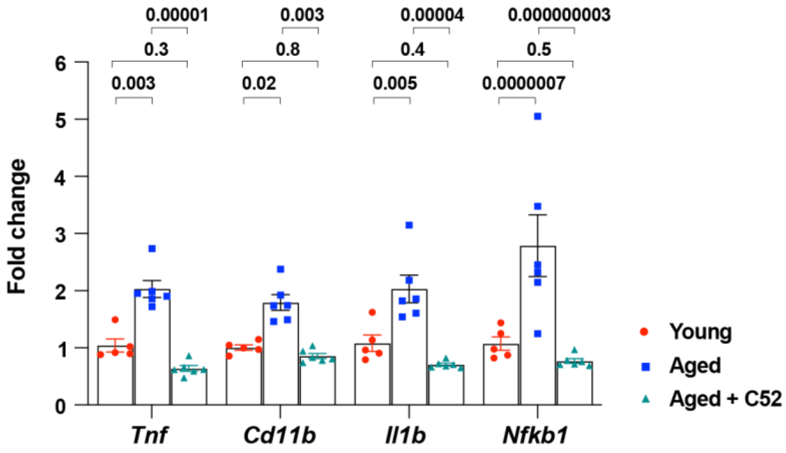


**Supplementary Fig. S1: Age-related neuroinflammation drives MT1-MMP activation in the mouse hippocampus**.

Transcript levels of pro-inflammatory genes including *Tnf, CD11b*, *Il1b, and Nfkb* in the hippocampus of young, aged, and aged + C52-treated mice (10 mg/kg/day for one month). Two-way ANOVA followed by Tukey's multiple comparisons test ($n = 5$ young, $n = 6$ aged, $n = 6$ aged + C52). Data are expressed as mean ± SEM, and each data point represents individual mice.

Supplementary Fig. S2

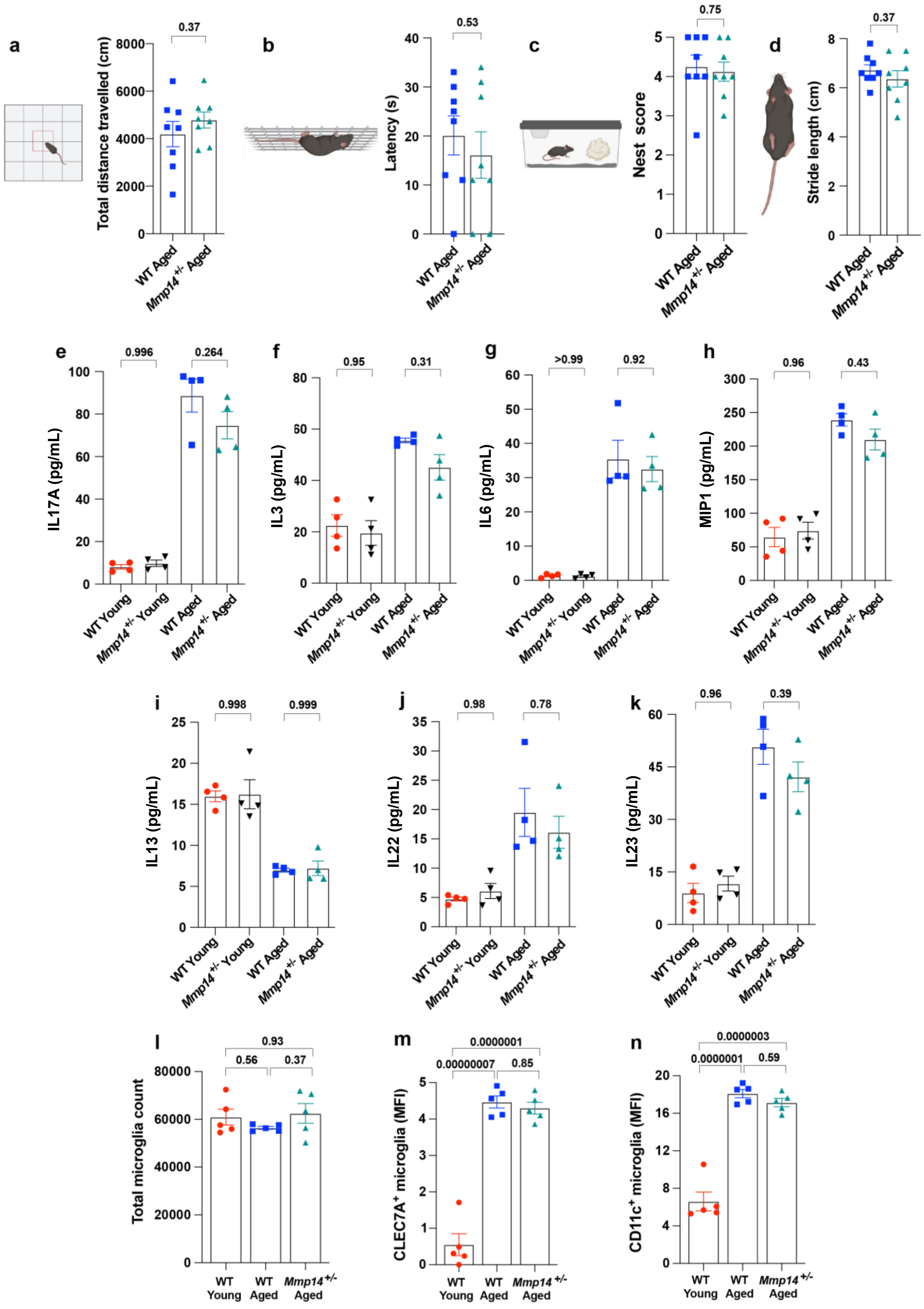

**Supplementary Fig. S2:** ***Mmp14*** **heterozygosity does not affect the physical activity of mice in ageing.**

**(a)** The total distance traveled in the open field test by aged WT and aged *Mmp14*$^{+/-}$ mice. Two-tailed unpaired Student's t-test (*n* = 8 mice per group). (**b)** The latency to fall during the hanging wire test. Two-tailed unpaired Student's t-test (*n* = 8 mice per group). (**c)** The cumulative nest score was calculated from the nest-building test. Two-tailed unpaired Student's t-test (*n* = 8 mice per group). (**d)** The stride length during the gait analysis. Two-tailed unpaired Student's t-test (*n* = 8 mice per group). (**e-k)** Levels of pro-inflammatory factors, including IL17A **(e)**, IL-3 **(f)**, IL6 **(g)**, MIP1 **(h)**, IL13 **(i)**, IL22 **(j)**, and IL23 **(k)** in hippocampal lysates obtained from young and aged WT and *Mmp14*$^{+/-}$ mice. One-way ANOVA followed by Tukey's multiple comparisons test (*n* = 4 mice per group). **(l-n)** Flow cytometry analysis from hippocampal lysates for **(e)** total microglia (CD11b + CD45 low) and **(m-n)** microglial activation. Quantifying mean fluorescent intensity (MFI) data for CLEC7A and CD11c-positive microglia. One-way ANOVA followed by Tukey's multiple comparisons test (*n* = 5 mice per group). Data are expressed as mean ± SEM, and each data point represents individual mice.

**Supplementary Fig. S3**

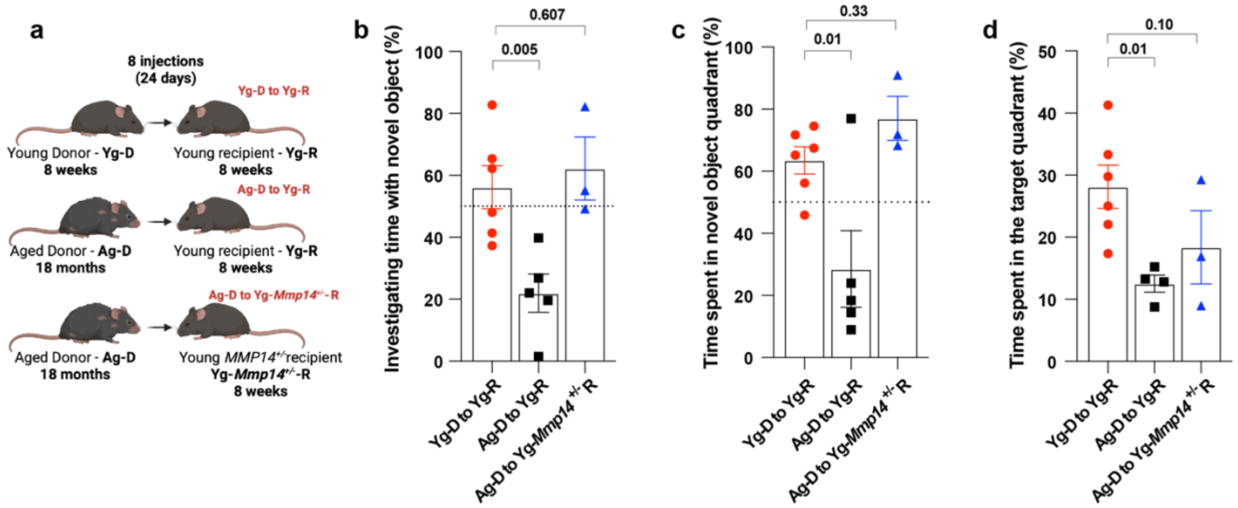


**Supplementary Fig. S3: *Mmp14* heterozygosity protects against cognitive impairment induced by blood transfusion from aged mice.**

**(a)** Schematic of the blood transfer from the donor (D) to the recipient (R) mice. **(b-c)** The investigating time with a novel object **(b)** and the time spent in the novel object quadrant **(c)** during the novel object recognition test by young recipient (Yg-R) and *Mmp14*$^{+/-}$ recipient (*Mmp14*$^{+/-}$R) mice injected with blood from young donor (Yg-D) or aged donor (Ag-D) mice. One-way ANOVA followed by Fisher's LSD post hoc test. ($n$ = 6 Yg-D to Yg-R, $n$ = 5 Ag-D to Yg-R, $n$ = 3 Ag-D to *Mmp14*$^{+/-}$R). **(d)** Percentage of total time spent in the target quadrant in the Barnes maze test. One-way ANOVA followed by Fisher's LSD post hoc test. ($n$ = 6 Yg-D to Yg-R, $n$ = 4 Ag-D to Yg-R, $n$ = 3 Ag-D to *Mmp14*$^{+/-}$R). Data are expressed as mean ± SEM, and each data point represents individual mice.

**Supplementary Fig. S4**

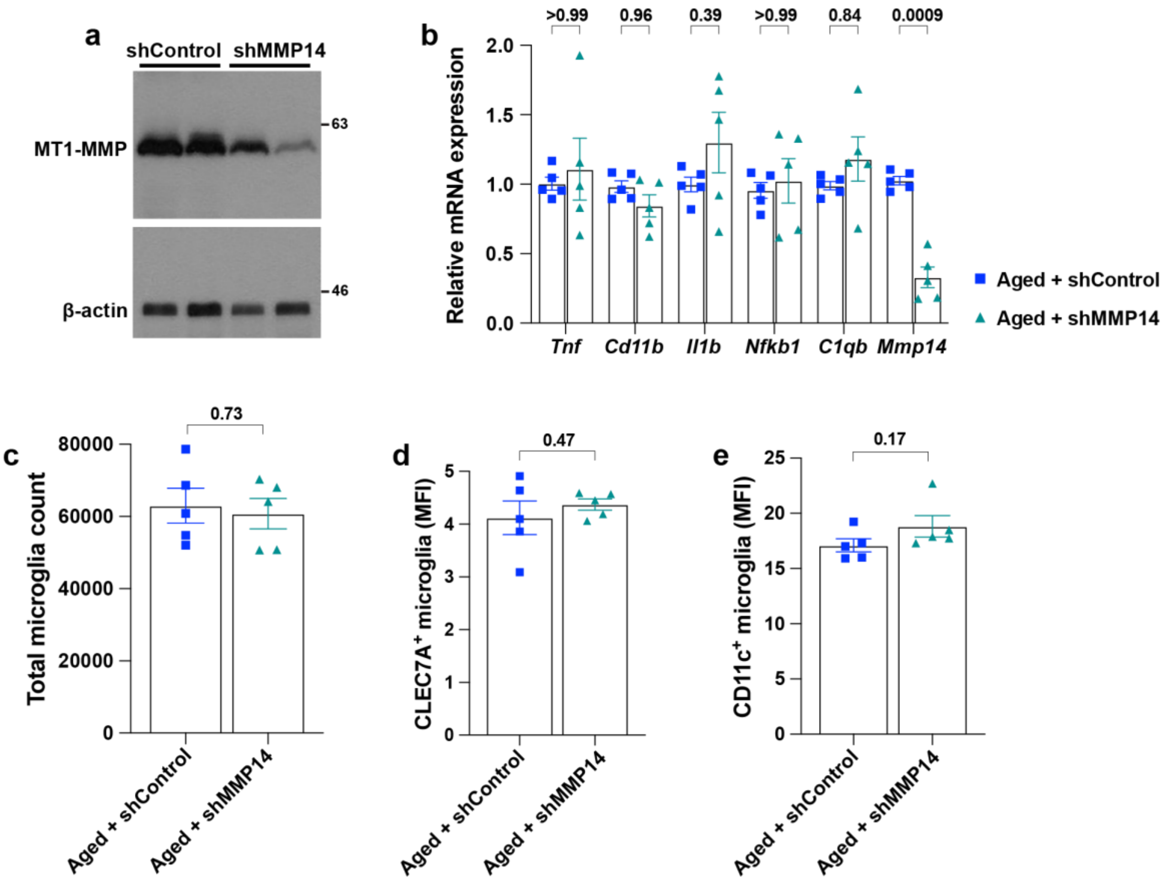


**Supplementary Fig. S4: Ablation of hippocampal MT1-MMP does not alter the age-associated neuroinflammation in mice.**

**(a)** Representative western blot of MT1-MMP from hippocampal lysates of mice injected with shControl or shMMP14 (n = 2 mice per group). **(b)** Transcript levels of pro-inflammatory genes, including *Tnf, Cd11b*, *Il1b*, *Nfkb1, C1qb,* and *Mmp14* in the hippocampus of aged mice injected with shControl or shMMP14. Two-way ANOVA followed by Šídák's multiple comparisons test (*n* = 5 mice per group). **(c-e)** Aged mice hippocampal lysates were examined for **(e)** total microglia (CD11b + CD45 low) and **(d-e)** microglial activation using flow cytometry. Quantifying MFI data for CLEC7A and CD11c positive microglia. One-way ANOVA followed by Tukey's multiple comparisons test (*n* = 5 mice per group). Data are expressed as mean ± SEM, and each data point represents individual mice.

**Supplementary Fig. S5**

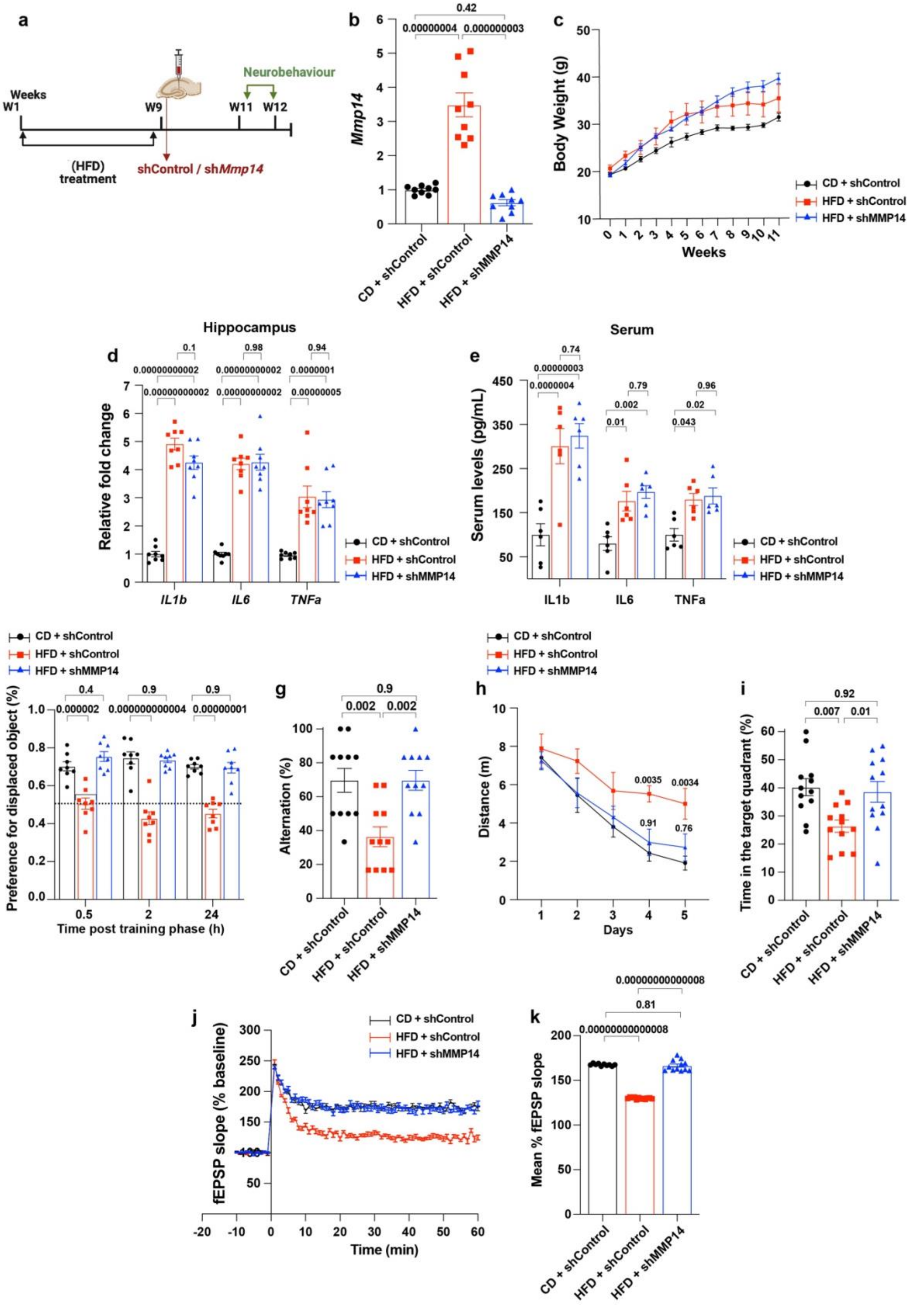

**Supplementary Fig. S5: Selective ablation of hippocampal MT1-MMP rescues memory deficits in obese mice.**

**(a)** Schematic of the experimental paradigm. **(b)** Transcripts levels of *Mmp14* in the hippocampus of mice fed with a CD or HFD and injected with shControl or shMMP14. One-way ANOVA followed by Tukey's multiple comparisons test (*n* = 9 mice per group). **(c)** Changes in the body weight were examined over eleven weeks. **(d)** Transcript levels of pro-inflammatory genes, including *Il1b, IL6, and TNF,* in the hippocampus of mice. One-way ANOVA followed by Tukey's multiple comparisons test (*n* = 8 mice per group). **(e)** Serum analysis for inflammatory markers. One-way ANOVA followed by Tukey's multiple comparisons test (*n* = 6 mice per group). **(f)** The object location task to examine the preference for the displaced object. Two-way ANOVA followed by Šídák's multiple comparisons test *(n* = 8 mice per group). **(g)** The Y maze test showed that spontaneous alternation was impaired in HFD-treated mice, whereas restored in HFD + shMMP14 treated mice. One-way ANOVA followed by Tukey's multiple comparisons test (*n* = 11 mice per group). **(h-i)** Morris water maze task. Distance traveled to reach the escape platform **(h)** and the time spent in the target quadrant **(i)**. Two-way ANOVA followed by Šídák's multiple comparisons test (h); One-way ANOVA followed by Tukey's multiple comparisons test (i) (*n* = 12 mice per group). **(j)** Changes in total excitatory postsynaptic potential (tEPSP) in the hippocampal CA1 for LTP analysis **(k)** and the average of fEPSP. One-way ANOVA followed by Tukey's multiple comparisons test (*n* = 8 for CD + shControl and *n* = 12 for HFD + shControl and HFD + shMMP14. Data are expressed as mean ± SEM, and each data point represents individual mice.

**Supplementary Fig. S6**

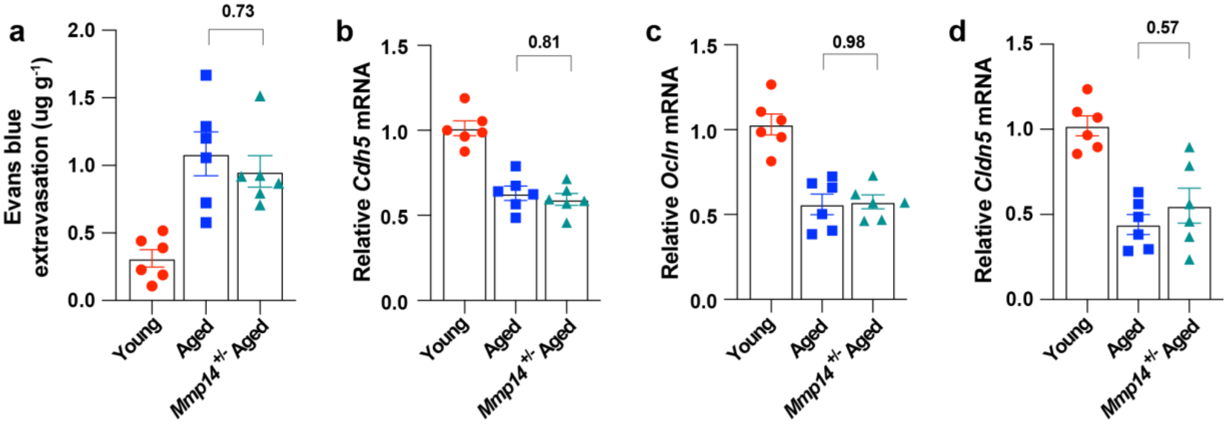


**Supplementary Fig. S6: *Mmp14* heterozygosity does not alter the integrity of the blood-brain-barrier in aged mice**

**(a)** Evans blue extravasation in young, aged, and *Mmp14*$^{+/-}$ aged mice. One-way ANOVA followed by Tukey's multiple comparisons test ($n$ = 6 mice per group). **(b-d)** Transcript levels of *Cdh5* **(b)**, *Ocln* **(c)**, and *Cldn5* **(d)** in the hippocampus. One-way ANOVA followed by Tukey's multiple comparisons test ($n$ = 6 mice per group). Data are expressed as mean ± SEM, and each data point represents individual mice.

**Supplementary Fig. S7**

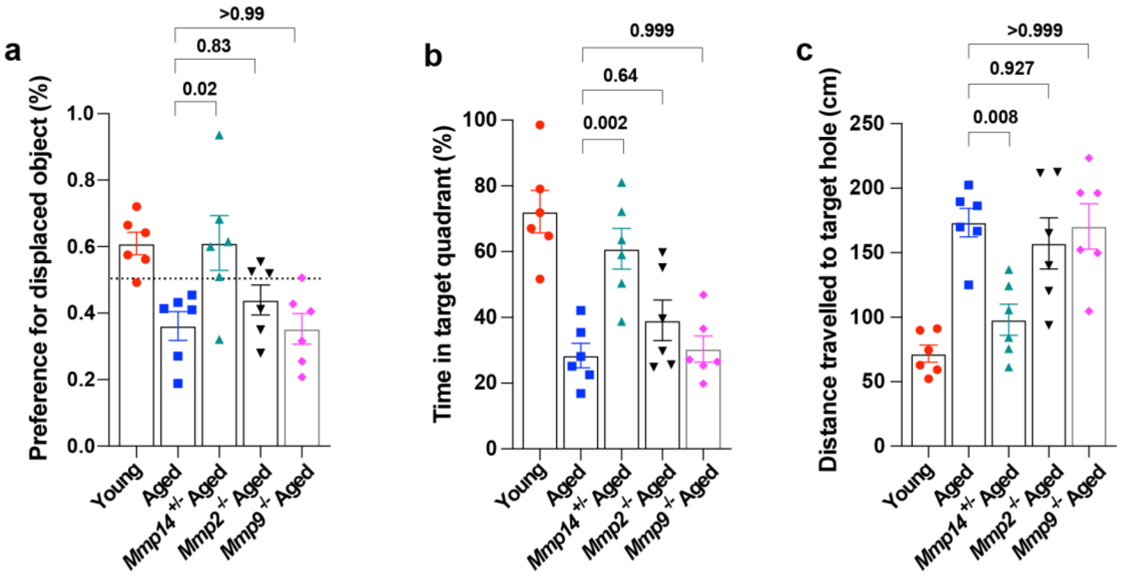


**Supplementary Fig. S7: Cognitive aging is specifically regulated by MT1-MMP.**
**(a)** The preference for the displaced object in the object location task by young, aged, *Mmp14*$^{+/-}$ aged, *Mmp2*$^{-/-}$ aged, and *Mmp9*$^{-/-}$ aged mice. One-way ANOVA followed by Tukey's multiple comparisons test ($n$ = 6 mice per group). (**b-c**) Percentage of the total time spent in the target quadrant **(b)** and distance traveled to the target hole **(c)** in the Barnes maze test. One-way ANOVA followed by Tukey's multiple comparisons test ($n$ = 6 mice per group). Data are expressed as mean ± SEM, and each data point represents individual mice.

**Supplementary Fig. S8**

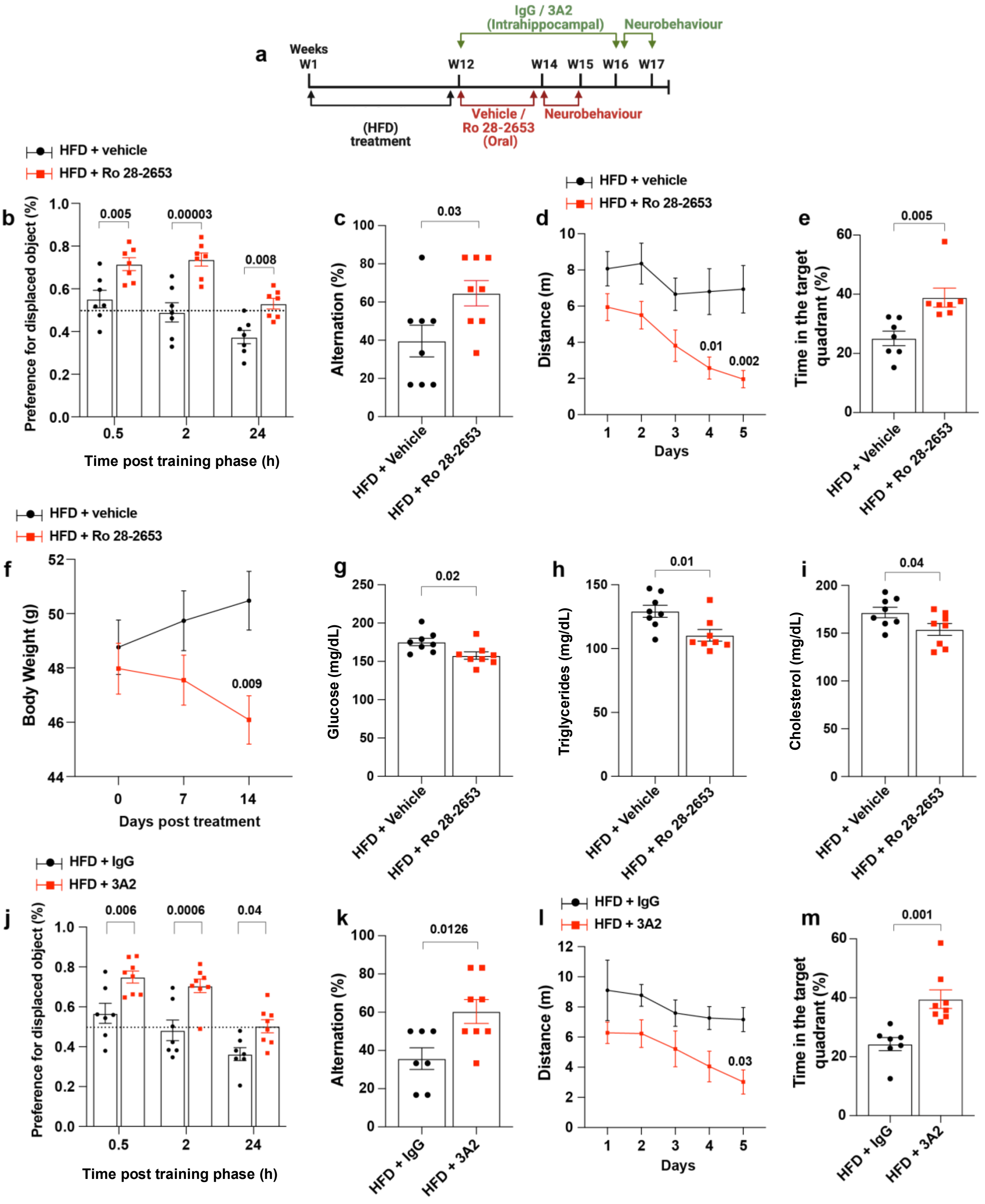

**Supplementary Fig. S8: Pharmacological inhibition of MT1-MMP improves cognitive function in obese mice.**

**(a)** Schematic of the experimental paradigm. Mice were fed either a CD or a HFD for twelve weeks. Subsequently, the mice were divided into four groups and received either vehicle or RO 28-2653 (50 mg/kg) treatment for two weeks, followed by cognitive function analysis. CD/HFD-fed mice were intrahippocampally injected with IgG or 3A2 (15 mg/kg) using an implanted osmotic pump for four weeks, followed by cognitive assessment. **(b)** The preference for the displaced object in the object location task. Two-way ANOVA followed by Šídák's multiple comparisons test *(n* = 7 mice per group). **(c)** Alteration % during the Y-Maze task. Two-tailed unpaired Student's t-test (*n* = 8 mice per group). **(d-e)** Morris water maze task. Distance traveled to reach the escape platform **(d)** and the time spent in the target quadrant **(e)**. Two-way ANOVA followed by Šídák's multiple comparisons test (d); Two-tailed unpaired Student's t-test (e) (*n* = 7 mice per group). **(f)** Body weight changes observed during the two-week RO 28-2653 treatment. Two-way ANOVA followed by Šídák's multiple comparisons test (*n* = 8 mice per group). **(g-i)** Serum analysis for glucose **(g)**, TG **(h)**, and total cholesterol **(i)** levels at the end of the study. Two-tailed unpaired Student's t-test (*n* = 8 mice per group). **(j-m)** Examination of the 3A2 effect on cognitive function performed using the **(j)** object location test (Two-way ANOVA followed by Šídák's multiple comparisons test, *n* = 7-8 mice per group), **(k)** Y-maze test (Two-tailed unpaired Student's t-test, *n* = 7-8 mice per group), and **(l-m)** Morris water maze task [Two-way ANOVA followed by Šídák's multiple comparisons test **(l)**; Two-tailed unpaired Student's t-test (m), *n* = 7 mice per group]. Data are expressed as mean ± SEM, and each data point represents individual mice.

**Supplementary Fig. S9**

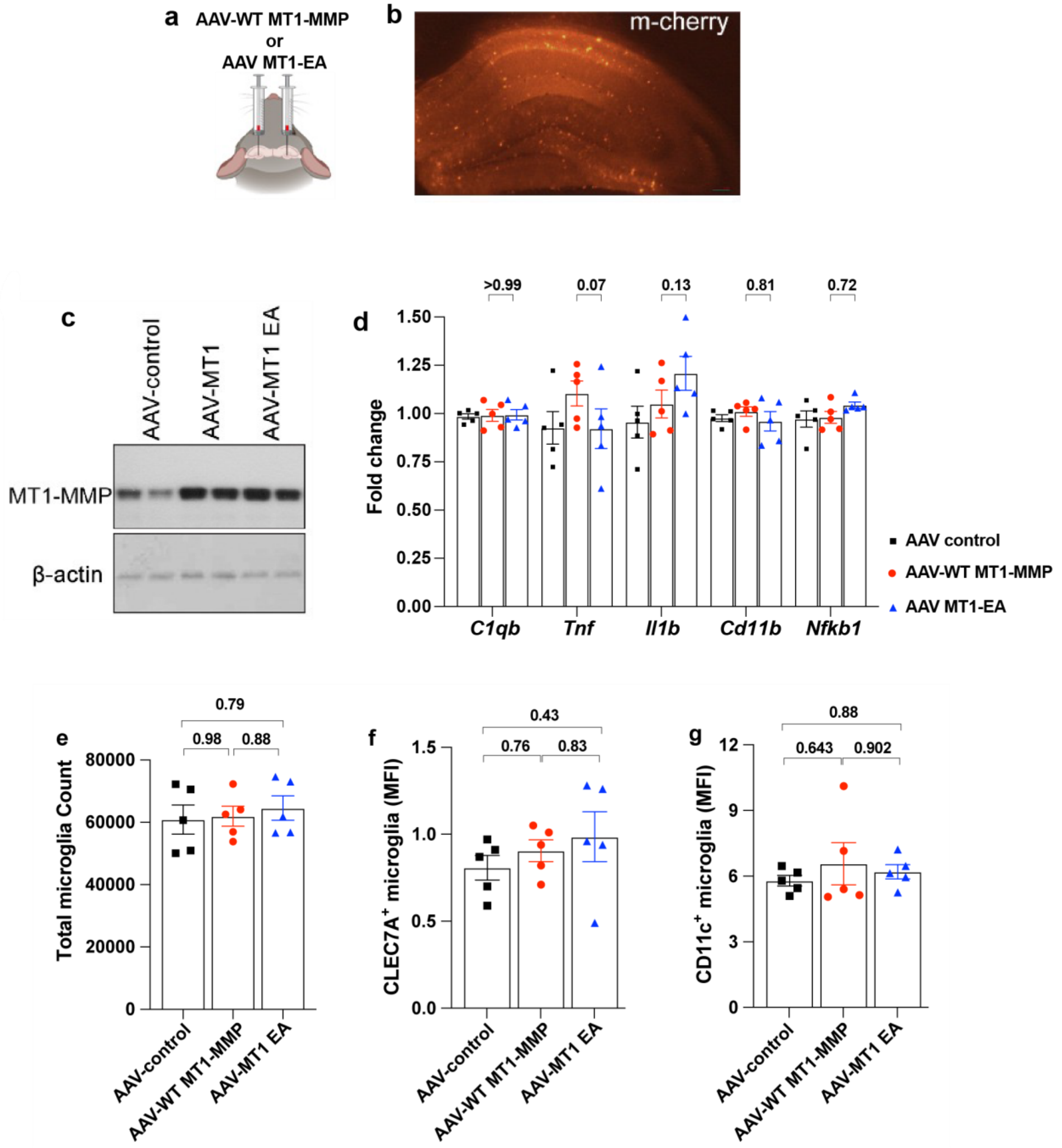


**Supplementary Fig. S9: Increasing MT1-MMP expression does not alter the inflammatory responses in the hippocampus.**

**(a)** Schematic of bilateral AAV injection in the DG region of the mice hippocampus. **(b)** Representative images show the successful transduction of AAV-control vectors labelled by mCherry in the hippocampal regions (mCherry-positive cells, red). **(C)** MT1-MMP expression levels in the hippocampus were examined by western blotting from mice receiving either AAV-control, AAV WT MT1, or AAV MT1 EA ($n$ = 2 mice per group). **(d)** mRNA levels of pro-

inflammatory genes including *C1qb, Tnf, Il1b, Cd11b* and *Nfkb1* in the mice hippocampus. Two-way ANOVA followed by Tukey's multiple comparisons test ($n$ = 5 mice per group). **(e-h)** Flow cytometry analysis from aged mice hippocampus for **(e)** total microglia (CD11b + CD45 low). Quantification data of MFI for CLEC7A and CD11c to examine microglia activation. One-way ANOVA followed by Tukey's multiple comparisons test ($n$ = 5 mice per group). Data are expressed as mean ± SEM, and each data point represents individual mice.

**Supplementary Fig. S10**

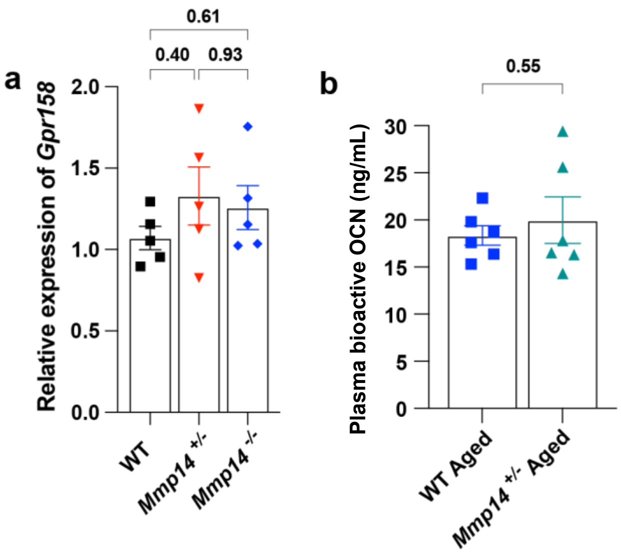


**Supplementary Fig. S10: MT1-MMP does not regulate the bioavailability of OCN.**
**(a)** The hippocampal mRNA level of *Gpr158* in WT, *Mmp14*$^{+/-}$, and *Mmp14*$^{-/-}$ mice. One-way ANOVA followed by Tukey's multiple comparisons test ($n$ = 5 mice per group). **(b)** The plasma level of OCN in aged and *Mmp14*$^{+/-}$ aged mice. Two-tailed unpaired Student's t-test ($n$ = 6 mice per group). Data are expressed as mean ± SEM, and each data point represents individual mice.

**Supplementary Fig. S11**

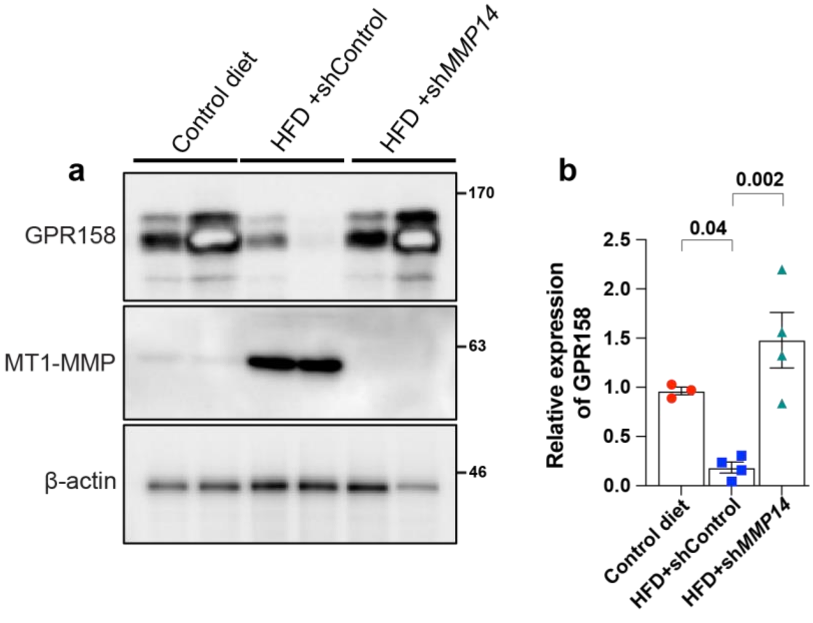


**Supplementary Fig. S11: MT1-MMP inhibits Gpr158 expression in obese mice.**
Western blot analysis was conducted to assess the protein expression levels of GPR158, MT1-MMP, and beta-actin in the hippocampus of mice subjected to either a CD or a HFD and treated with shControl or shMMP14.

**Supplementary Fig. S12**

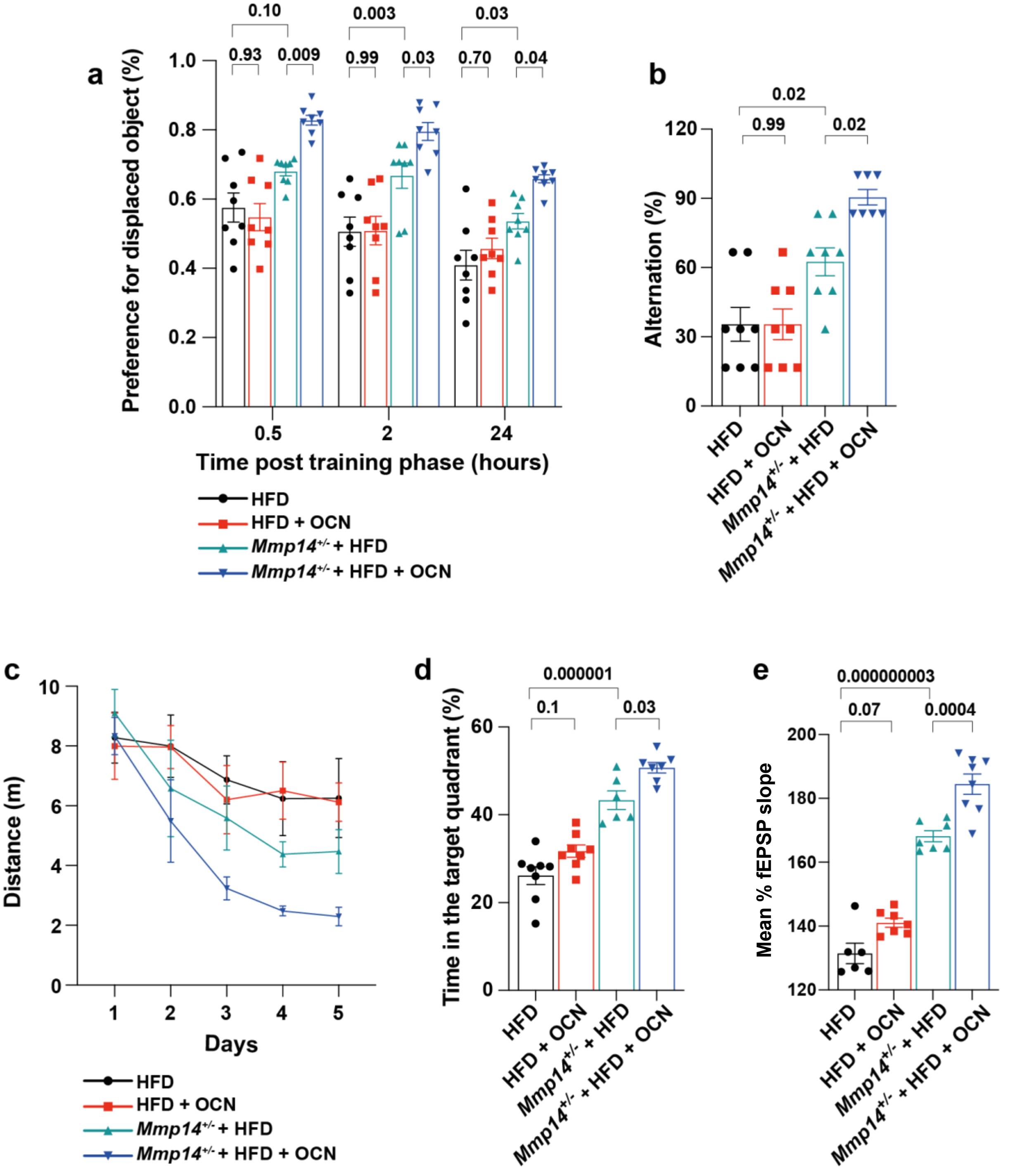


**Supplementary Fig. S12: MT1-MMP depletion restores the cognition-promoting effect of OCN in obese mice**

WT or *Mmp14*$^{+/-}$ were fed with HFD for twelve weeks and subsequently treated with rOCN **(a)** The preference for the displaced object in the object location task. Two-way ANOVA followed by

Šídák's multiple comparisons test *(n* = 8 mice per group). **(b)** Alteration % during the Y-Maze task. One-way ANOVA followed by Tukey's multiple comparisons test (*n* = 7-8 mice per group). **(c-d)** Morris water maze task. Distance traveled to reach the escape platform **(c)** and the time spent in the target quadrant **(d)**. One-way ANOVA followed by Tukey's multiple comparisons test (*n* = 6-8 mice per group). **(e)** Changes in fEPSP in the hippocampal CA1 for LTP analysis. One-way ANOVA followed by Tukey's multiple comparisons test (*n* = 6-8 mice per group). Data are expressed as mean ± SEM, and each data point represents individual mice.

**Supplementary Table S1**

| Gene | Forward (5'-3') | Reverse (5'-3') |
|---|---|---|
| ***C1qb*** | CAACCAGGCACTCCAGGGATAA | CCAACTTTGCCTGGAGTCCCAG |
| ***Cd11b*** | TACTTCGGGCAGTCTCTGAGTG | ATGGTTGCCTCCAGTCTCAGCA |
| ***Cdh5*** | GAACGAGGACAGCAACTTCACC | GTTAGCGTGCTGGTTCCAGTCA |
| ***Cldn5*** | TGACTGCCTTCCTGGACCACAA | CATACACCTTGCACTGCATGTGC |
| ***Gapdh*** | AACATCCATCGCGGTCTC | CCATTTTGTCTACGGGACGA |
| ***Gpr158*** | GCTCATCCTGTTGGAAACAAT | TGGCTCAAAGTACAGAATAACGAC |
| ***Il1b*** | TGGACCTTCCAGGATGAGGACA | GTTCATCTCGGAGCCTGTAGTG |
| ***Mmp14*** | ATGTCTCCCGCCCCTCGACC | TGGGTACGCAGGTCCCCTGG |
| ***Nfkb1*** | GCTGCCAAAGAAGGACACGACA | GGCAGGCTATTGCTCATCACAG |
| ***Ocln*** | TGGCAAGCGATCATACCCAGAG | CTGCCTGAAGTCATCCACACTC |
| ***Tnf*** | GGTGCCTATGTCTCAGCCTCTT | GCCATAGAACTGATGAGAGGGAG |

**Supplementary Table S1: qPCR primers sequence information**
